# An optimal antibiotic selection framework for Sepsis patients using Artificial Intelligence

**DOI:** 10.1038/s41746-024-01350-y

**Published:** 2024-11-29

**Authors:** Philipp Wendland, Christof Schenkel-Häger, Ingobert Wenningmann, Maik Kschischo

**Affiliations:** 1 University of Applied Sciences Koblenz, Department of Mathematics and Technology, Remagen, 53424 Germany; 2 University of Applied Sciences Koblenz, Department of Economics and Social Studies, Remagen, 53424 Germany; 3 University Hospital Bonn, Department of Anesthesieology and Operative Intensive Care Medicine, Bonn, 53127 Germany; 4 University of Koblenz, Department of Computer Science, Koblenz, 56070 Germany

**Keywords:** Bacterial infection, Computational science, Adverse effects, Predictive medicine, Antimicrobial therapy

## Abstract

In this work we present OptAB, the first completely data-driven online-updateable antibiotic selection model based on Artificial Intelligence for Sepsis patients accounting for side-effects. OptAB performs an iterative optimal antibiotic selection for real-world Sepsis patients focussing on minimizing the Sepsis-related organ failure score (SOFA-Score) as treatment success while accounting for nephrotoxicity and hepatotoxicity as serious antibiotic side-effects. OptAB provides disease progression forecasts for (combinations of) the antibiotics Vancomycin, Ceftriaxone and Piperacillin/Tazobactam and learns realistic treatment influences on the SOFA-Score and the laboratory values creatinine, bilirubin total and alanine-transaminase indicating possible side-effects. OptAB is based on a hybrid neural network differential equation algorithm and can handle the special characteristics of patient data including irregular measurements, a large amount of missing values and time-dependent confounding. OptAB’s selected optimal antibiotics exhibit faster efficacy than the administered antibiotics.

## Introduction

Sepsis is defined as a severe dysregulation of the immune system’s response to an infection that damages its own healthy tissue and organs. Sepsis often leads to organ failure or even death. Sepsis is one of the most severe problems facing healthcare systems worldwide with 48.9 million cases and 11 million deaths representing 19.7% of all global deaths in 2017^1,2^. Reports in the UK and Australia identify Sepsis as a leading cause of avoidable deaths^3,4^. Bacterial infections cause 80–90% of all Sepsis cases^5–7^. Besides early detection the selection of the correct initial antibiotic(s) combating the sepsis causing pathogens is one of the main challenges in the treatment of Sepsis due to the variety of different pathogens.

There are many different recommendations for the selection of appropriate initial antibiotic(s) for Sepsis based on different sites of infection, severity of symptoms, and risks of antibiotic-resistant pathogens^8,9^. The results of microbiological cultures including tests for antibiotic resistances are of great importance for the choice of antibiotics, but in 30–70% of all Sepsis patients no pathogen can be detected^10–12^. Even if bacterial pathogens can be detected, often several antibiotics are sensitive and (as in our datasets) the results of microbiological cultures are often only available with a delay of two or three days. In such scenarios, where microbiological cultures are unavailable at the outset, the choice of initial antibiotics is challenging. It is advisable to opt for broad-spectrum antibiotics in these situations^13^, but it is not clear whether combinations of two antibiotics or a single antibiotic should be administered^9,14^. A more individualized selection of antibiotics, supported by data-driven methods, could potentially decrease mortality, severity of Sepsis, drug-induced side-effects and accelerate the recovery process.

Current research on Sepsis management with machine learning and artificial intelligence usually focuses on the early detection or the disease progression of Sepsis, while data-driven selection of antibiotics is largely overlooked^15–19^. Although the recently published T4 algorithm adresses the search for an optimal timing of antibiotic administration for Sepsis patients, it does not differentiate between different antibiotics and corresponding side-effects^20^. The so-called Artificial Intelligence clinician learns optimal treatment strategies of intraveneous fluids and vasopressors for Sepsis, but does not incorporate antibiotics or side effects into its treatment optimization^21^.

**Fig. 1:**
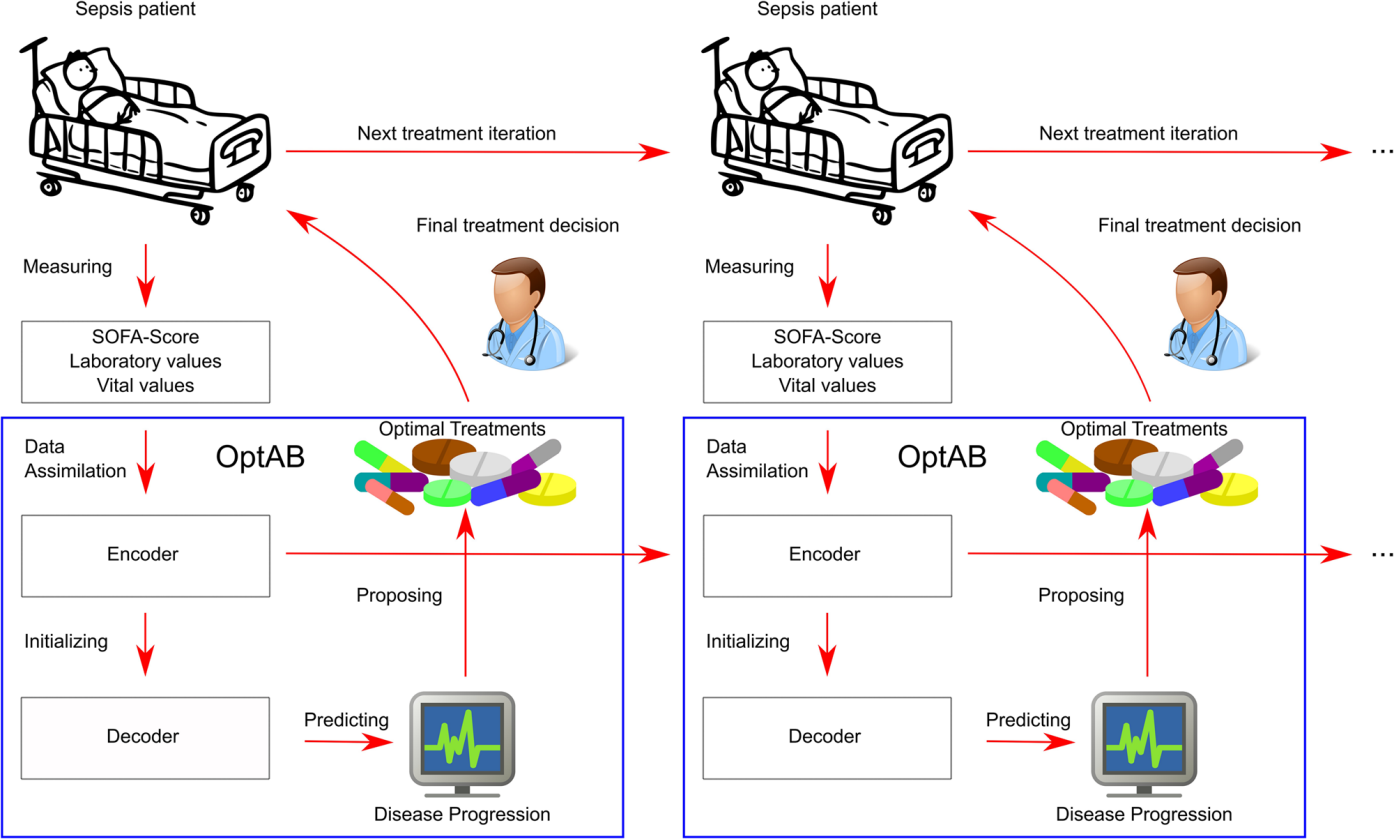
Outline of the online-updateable optimal treatment selection model OptAB. OptAB iteratively selects optimal antibiotic treatments for Sepsis patients by minimizing the SOFA-score corresponding to treatment success, while accounting for drug-associated side-effects. At each iteration all available measurements are fed into OptAB to obtain updated predictions. Additionally, OptAB provides predictions of the SOFA-Score and laboratory values indicating contraindications and side-effects for all treatment options. We used cliparts from anonymous artists published under a free license at openclipart.org and cocomaterial.com for this Figure.

We created OptAB, to the best of our knowledge the first data-driven model for optimal antibiotic selection with Artificial Intelligence (AI). OptAB assimilates data from the Sepsis-related organ failure score (SOFA-Score) as the most important measure for the disease state together with demographic data, vital values and laboratory values to forecast the future disease course under different antibiotic treatments (see Fig. 1). This enables clinicians to compare the predictions for an individual patient under different choices of antibiotic(s) over time.

The severity of Sepsis is assessed using the SOFA-Score, which consists of six subscores with levels from zero to four that describe the condition of the central nervous system, the cardiovascular system, the respiratory system, coagulation, liver and kidney^22^. For each subscore, a moving average is calculated based on the worst measured value of the last 24 h. OptAB forecasts the SOFA-Score as a continuous variable to allow for a better comparison of its trend (see Supplementary material for a detailed definition of the SOFA-Score). The primary goal of optimal antibiotic therapy is to treat the pathogenic microorganism, with the reduction of the SOFA score reflecting treatment success, while also accounting for contraindications by forecasting laboratory values that indicate antibiotic-associated side effects. These side effect forecasts can complement existing guidelines and recommendations when certain antibiotics should not be administered. Our focus was on patients in the Intensive Care Unit (ICU), as the SOFA score is primarily calculated from data collected in an ICU or a comparable setting.

Clinical routine data are often measured irregularly and exhibit many missing values. Laboratory values such as platelets and vital values like blood pressure are not only measured at various times, but also at different times for each patient in relation to Sepsis onset. As a consequence a disease progression forecast model needs to handle irregularly sampled data, incorporate new measurements (online-updateable data assimilation) and impute missing values. A particular challenge for the estimation of treatment effects in observational data is time-dependent confounding. An important source of confounding is that individual treatment decisions made by clinicians are based on diagnoses, vital values and laboratory values as well as their experience and knowledge. This causes statistical dependencies between these time-varying covariates and treatment decisions, which can lead to bias in treatment effect estimates^23,24^.

OptAB is based on the Treatment-Effect Controlled Differential equation (TE-CDE) as a state-of-the-art online-updateable disease forecast model to handle the special properties of patient data including time-dependent confounding^25^. Here, we extend and test the TE-CDE approach to optimize dynamic treatment regimes in a realistic medical setting using real-world patient data.

OptAB suggests optimal antibiotic treatments by minimizing the SOFA-score corresponding to treatment success while accounting for drug-associated side-effects. A reliable assessment of the efficacy of the initial treatment is only possible 48 h after treatment. The treatment should remain unchanged during this initial phase, except for incoming results of microbiological cultures, which can provide a more detailed picture of the pathogen situation, as well as updated resistance tests and laboratory values indicating severe contraindications. After the first evaluation and potential treatment adjustment, efficacy should be re-evaluated every 24 h^9,26,27^. We mimic this practice in OptAB. The first forecast is based on the available data at Sepsis onset. At every evaluation of the efficacy or change of treatment, we feed all available measurements into OptAB to get updated forecasts (see Fig. 1). If the SOFA-Score is decreasing over a period of 48 h, common guidelines recommend to de-escalate antibiotic treatment^9,26^. If a fungal or a viral pathogen is detected in a microbiological culture, OptAB notifies the attending physician.

Given the life-threatening condition of sepsis patients, the primary goal of treatment is to effectively combat the sepsis, which in turn leads to a reduction in the SOFA score. However, administration should be avoided if an antibiotic is contraindicated or, if different treatments demonstrate similar effectiveness, the one with the least side effects should be preferred. To aid in this decision-making, OptAB provides forecasts of the SOFA score and laboratory values indicating potential antibiotic-associated side effects, particularly those related to renal and kidney function. This offers physicians a more comprehensive assessment of treatment options tailored to individual patients. OptAB iteratively suggests antibiotics (or combinations of two antibiotics) that are predicted to minimize the SOFA score after 24 or 48 h, while adhering to thresholds for antibiotic-specific contraindications and side effects. When these thresholds are exceeded, OptAB raises warnings to identify high-risk patients and propose alternative treatments. For a more detailed explanation of the mathematical framework behind OptAB, please refer to the Methods section.

The main contributions of our paper are the following:

- We created an online-updateable disease progression forecasting model for Sepsis based on the TE-CDE^25^, which is able to handle the special characteristics of patient data including irregular measurements, many missing values and time-dependent confounding.
- We developed OptAB, the first online-updateable optimal antibiotic selection model based on Artificial Intelligence to maximize treatment success while accounting for contraindications and side-effects.
- We performed an optimal antibiotic selection with OptAB for real-world Sepsis patients focussing on minimizing the SOFA-Score as treatment success and nephrotoxicity and hepatotoxicity as severe side-effects.

The report is based on the SRQR reporting guidelines (see Supplementary Table 2)^28^.

## Results

We trained and tested OptAB on the electronic health record (EHR) dataset MIMIC-IV^29,30^ containing over 450.000 admissions to the Beth Israel Deaconess Medical Center of the Harvard medical School including demographics, laboratory values, vital values, medications and diagnosis data. For an independent test set, we compared the disease progression of each individual patient to OptAB’s predictions under the same treatment regime as actually received (factual treatment). In addition, we used OptAB to predict disease courses under alternative treatments. We refer to these predictions of the disease progression under alternative and unobserved treatments as counterfactuals.

We focus on the three first-line antibiotics Vancomycin, Ceftriaxone and Piperacillin-Tazobactam recommended for the treatment of Sepsis, where at least 5000 patients of the 26,111 Sepsis patients of the MIMIC-IV dataset are treated with. Data cleaning and patient selection resulted in a training set of 2842 and a test set of 711 Sepsis patients, see the Methods section for details.

Vancomycin is effective against a broad spectrum of gram-positive pathogens, including MRSA^31^. Unfortunately, Vancomycin is renal toxic and can cause acute kidney injuries^32^ and Ceftriaxone is hepatotoxic and can lead to severe liver injuries^33,34^. To incorporate the current recommendations, we implemented warnings into OptAB against treating patients diagnosed with a creatinine value higher than 2 mg/dl with Vancomycin or (at least) Stage-1 Acute Kidney Injury. According to current guidelines^35^ Stage-1 Acute Kidney Injury was defined as either an increase in creatinine of 0.3 mg/dl within 48 h, or an increase of 1.5 times the baseline creatinine within the previous 7 days or an urine output of less than 0.5 ml/kg/h for 6–12 h. Additionally, OptAB warns against administrating Ceftriaxone to patients with elevated alanine transaminase values exceeding 280 U/L indicating toxic liver injuries and liver cell damages or increased bilirubin total values higher than 2.4 mg/dl for men and 2.2 mg/dl for woman indicating jaundice and bile duct inflammation (see Methods for detailed information). These values can be adjusted to meet current guidelines or individual assessments by the physician.

As an external validation, we tested the OptAB model trained on the MIMIC-IV data set on AmsterdamUMCdb containing 23.106 admissions from 2003 to 2016 to the intensive care unit of the Amsterdam University Medical center including detailed EHR data^36^. For ease of exposition, these tests are provided in the Supplementary material.

### OptAB predicts the SOFA-Score together with laboratory values indicating side-effects over time

**Fig. 2:**
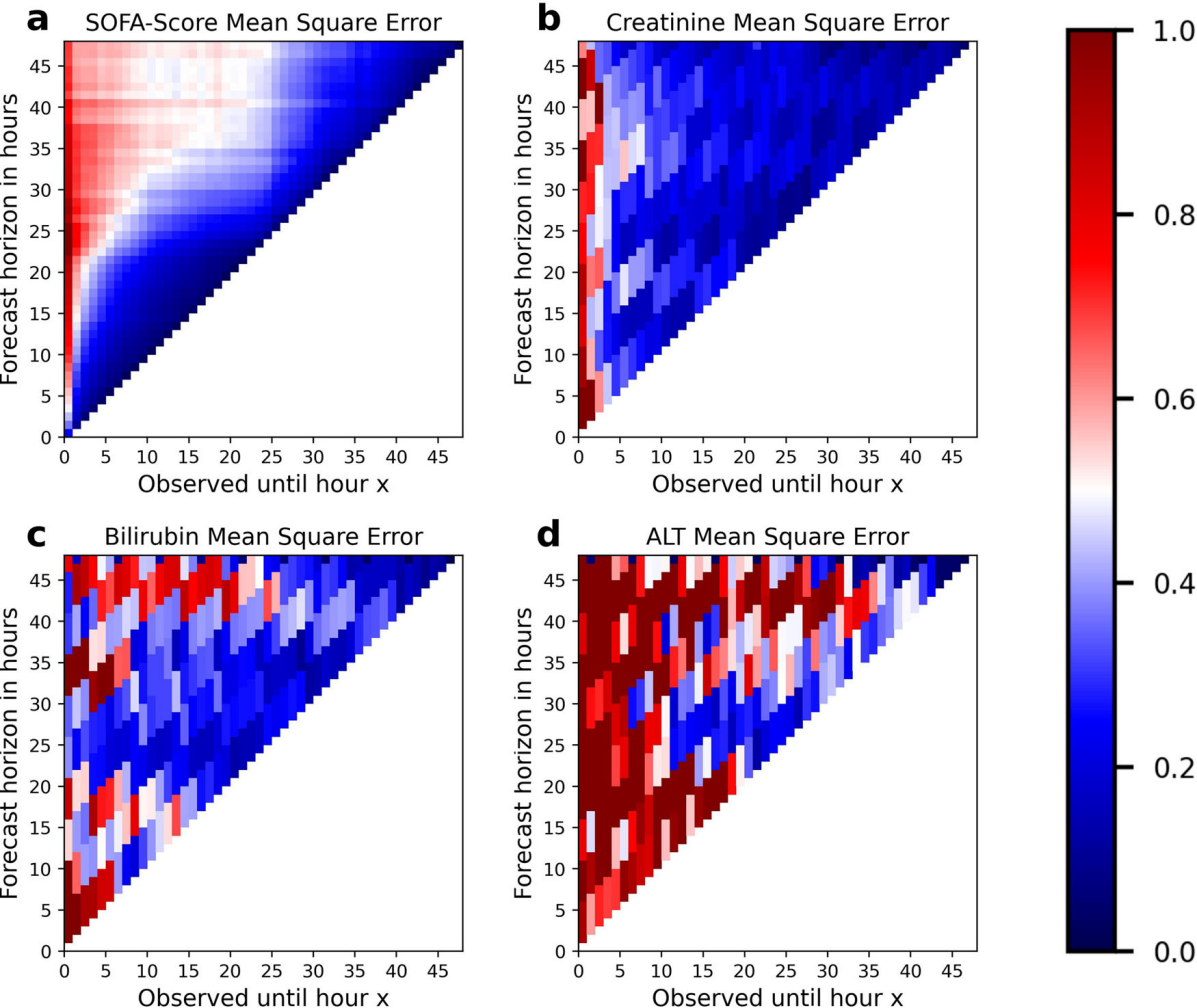
Accuracy of OptAB’s forecasts of the SOFA-Score and laboratory values indicating side effects. The heatmaps display the mean squared error (MSE) of OptAB's forecasts of the SOFA-Score (**a**), creatinine (**b**), bilirubin total (**c**) and alanine transaminase (ALT) (**d**). On both axes, time zero corresponds to Sepsis onset. The observation time on the x-axis is defined as the time span for which the patient data were assimilated into OptAB, before the forecast is made. The forecast horizon on the y-axis is the time span for which the forecast is made into the future. MSE-values are given in units of variance over all observations for the respective variable. For creatinine, bilirubin total and ALT, we averaged the MSE over 5 consecutive observation time points to account for sparser measurements.

To assess OptABs average forecast accuracy, we estimated the mean squared error (MSE) of its forecasts scaled in units of variance for a test set of 711 patients not used for training (Fig. 2). The accuracy depends on both the observation time and the observation horizon. The observation time is defined as the length of the time window for which the observed data were assimilated into OptAB until the forecast is made. The forecast horizon is the maximal time interval for which the prediction into the future is provided.

At sepsis onset (time zero), the SOFA score can be forecast by OptAB for the next hour with a MSE of less than 25% (Fig. 2a). SOFA score forecasts for more than one hour are less accurate due to the limited data availability for data assimilation right at sepsis onset. Observing the patient for at least one hour after Sepsis onset facilitates forecasts for the next 4–5 h with MSE smaller than 15%. Assimilating further data from ongoing patient monitoring enables predictions of the SOFA-Score for the next hour with MSEs <2–4% and increases the forecast horizon with MSE <15% to 10–15 h. After an observation time of approximately 15–20 h, the prediction horizon with MSE <15% shrinks to 5–7 h, which might reflect that many patients are at a critical point where the uncertainty about the future disease course is so high that predictions are intrinsically difficult, given the data available to OptAB.

In comparison to the SOFA score, the number of observations per patient and time interval is much lower for the side effect indicating laboratory values creatinine, bilirubin total and alanine transaminase. In the test cohort, often fewer than 30 recordings per hour are available for these laboratory values. Amongst the 711 patients in the test data set, there are 404 patients without any bilirubin total and 399 without any alanine transaminase recordings. For the remaining patients we have averaged the MSE for these three lab values over five consecutive time points to decrease the variability of the MSE estimates due to much larger observation intervals.

Assimilating data for at least four hours after sepsis onset substantially improves the accuracy of creatinine forecasts. After 15 h, predictions with a MSE of 15–30% are possible for a long forecast horizon (Fig. 2b). Bilirubin total forecasts are typically less accurate and the observation time to reach MSE-values less than 20% in units of the variance is around 17 h (Fig. 2c) with a shorter forecast horizon than for creatinine.

The MSEs of alanine transaminase (Fig. 2d) are typically much higher than those of the other variables, often exceeding 50% or more. Most of the alanine transaminase values range between 0 IU/L and 500 IU/L, but a few patients reach levels above 5000 IU/L. These extreme values have a large effect on the MSE. This is in line with previous reports that alanine transaminase levels are highly variable, both between repeated measurements of the same patient and between patients^37,38^.

Overall, OptAB is able to forecast the time course of the SOFA-score and creatinine with sufficient accuracy given a realistic observation time window after sepsis onset. Forecasting bilirubin total is slightly more difficult, presumably because of the small number of observations in both training and test sets. Extreme values of alanine transaminase for a small number of patients aggravate inaccuracies due to data sparsity for this laboratory value.

### OptAB learns realistic treatment effects

Forecasting the factual disease courses alone is not sufficient for treatment optimization. OptAB can predict the effect of different potential antibiotic treatments on the SOFA score and the side effect indicators. However, it is impossible to evaluate these predictions retrospectively using our real-world test data. Each patient received just one factual treatment and there are of course no observations for the counterfactual treatments. To still check whether the effects of different counterfactual treatments are plausible, we investigated, whether known toxic side effects of the different antibiotics are reflected by the counterfactual predictions. The rationale behind this is that treatment with a renal toxic antibiotic like Vancomycin should often lead to higher creatinine values than treatment with Ceftriaxone. At the same time, the laboratory values bilirubin total and alanine transaminase should often be higher under Ceftriaxone treatment compared to Vancomycin, due to the known hepatotoxic effect of Ceftriaxone. The individual effect can be different for some individual patients, because the Sepsis disease course itself might have an effect on these laboratory values. However, we expect at least to see a trend into the direction consistent with the known side effect profile of the different antibiotics.

**Fig. 3:**
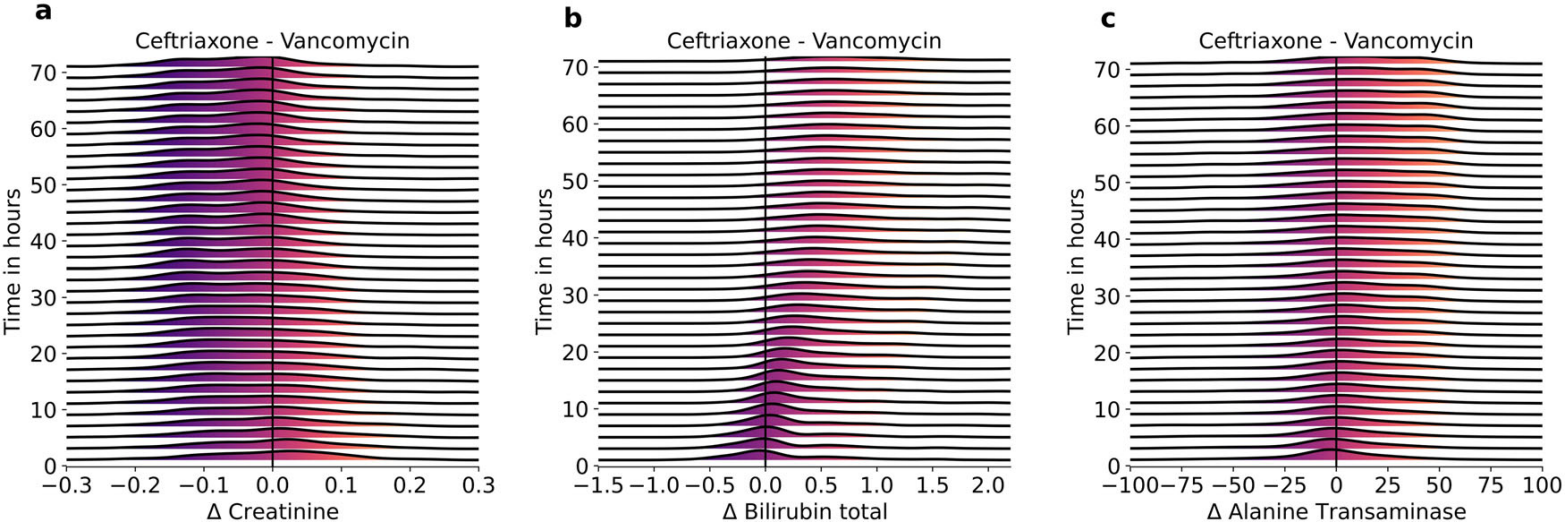
OptAB predicts plausible side effects under simulated treatments. OptAB was used to predict laboratory values indicating side effects in response to treatment with Vancomycin versus Ceftriaxone. For each patient, the time courses of the laboratory values under both alternative treatments were predicted by OptAB for a forecast time horizon of 72 h. Then, the difference Δ between the laboratory values under Vancomycin and Ceftriaxone treatment was taken for each patient. Time zero indicates the time point of the first treatment (both for the factual and the simulated). The density estimates indicate the temporal evolution of these differences for (**a**) creatinine (Δ Creatinine) values, (**b**) bilirubin total (Δ Bilirubin total) values and (**c**) alanine transaminase (Δ Alanine Transaminase).

We sampled individual patients from the test set (see Methods) and used OptAB to predict the time courses of the side effect indicating laboratory values in reponse to treatment with Vancomycin and Ceftriaxone for each patient. These simulated treatments were initialized at the same time point as the factual treatment of the individual patient and correspond to time zero in Fig. 3. Then we took the difference ΔCreatinine between the two creatinine time courses under Vancomycin versus Ceftriaxone treatments. Figure 3a displays the temporal evolution of the density estimates for Δ Creatinine. We proceeded in an analogous way with bilirubin total (Δ Bilirubin total) and alanine transaminase (Δ Alanine Transaminase), see Fig. 3b, c.

After an initial transient, the densities show the expected tendencies. Patients treated with Vancomycin tend to have higher creatinine levels and lower bilirubin total and alanine transaminase levels after some hours of treatment than patients treated with Ceftriaxone. The broad distributions, which can even develop different maxima and minima in the course of treatment, indicate individual effects on these laboratory values caused by different Sepsis disease courses.

### Discrepancies between OptAB’s predictions for similar patients are smaller than patient to patient variability

Unknown or hidden confounders in observational data can bias treatment effect estimates. To assess whether these confounders affect predictions under OptAB’s optimal treatments, we employed a matching approach. We compared OptAB’s predicted SOFA-Score progressions under its recommended optimal treatment with the factual SOFA-Scores of similar patients, who were factually treated with the same treatment (counterfactual matching).

**Fig. 4:**
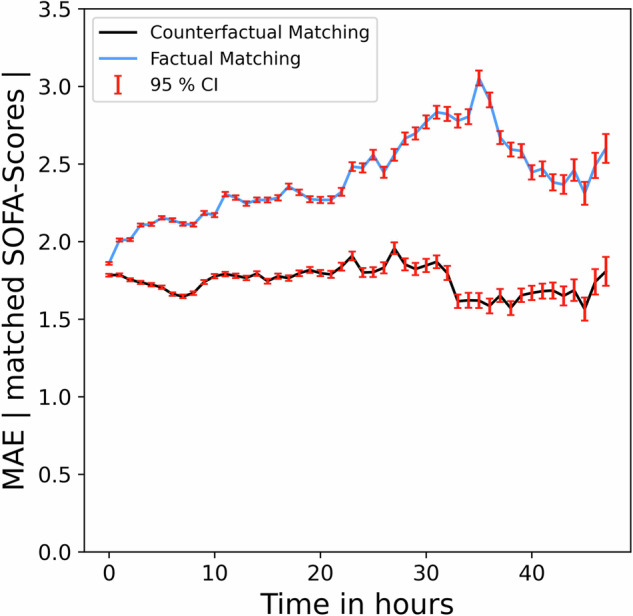
Average absolute difference of SOFA-Scores between matched patient pairs. For each patient, we compared OptAB's predicted SOFA-Score progression under its proposed optimal treatment with the factual SOFA-Scores of the most similar patient, who was factually treated with the same treatment (counterfactual matching). The euclidean distance between all covariates at Sepsis onset was used as the similarity measure. The black line shows the average absolute difference in SOFA-scores for the Counterfactual Matching. Moreover, we matched each patient to the most similar patient, who was factually treated with the same treatment (factual matching). The blue line shows the average absolute difference for the Factual Matching. The bars represent 95%-confidence intervals.

We first identified patients whose actual antibiotic treatment differed from OptAB’s proposed regimen, focusing on those treated within three hours of sepsis onset due to the timing’s impact on disease progression. For each patient, we computed the euclidean distance between their normalized covariables at sepsis onset and those of patients who were factually treated with the treatment proposed by OptAB. From these pairs of patients with the same counterfactual / factual treatment we selected the pairs with smallest euclidean distance at sepsis onset. As a result we obtained a set of counterfactually matched patient pairs which are similar at sepsis onset and the factual treatment of one patient is identical to the optimal counterfactual treatment of the other. For these matched pairs, we calculated the absolute difference of their SOFA-Scores and averaged this across all counterfactually matched pairs (Fig. 4.)

Even those counterfactually matched pairs of similar patients can differ in their disease progression, because they are not identical. To assess, how much of the differences in SOFA-Score predictions can be attributed to patient variability rather than prediction errors, we formed a second set of matched patient pairs. We matched each patient to the most similar patient, who was factually treated with the same treatment (Factual matching). We then compared the factual SOFA-Scores between these matched patients using the same methodology as described above (See Fig. 4).

The average absolute difference in SOFA-Scores for the counterfactual matching ranges between 1.5 and 2.0 during the first treatment iteration of 48 h. The average absolute difference stays relatively constant and does not increase over time. This indicates that OptAB’s SOFA-Score predictions under its proposed optimal treatment exhibit similar dynamics to the factual SOFA-Score time course of the matched patients. The slight differences at treatment initialization are presumably induced by patient variability among the matched pairs. The average absolute difference in SOFA-Scores for the factual matching is even higher, ranging between 1.8 and 3.1. This difference can be completely attributed to patient variability as we compared the factual SOFA-Scores in this case. Therefore, a large proportion of the differences in SOFA-Scores observed in counterfactual matching can be attributed to patient variability rather than prediction errors. These results suggest, that OptAB’s disease progression predictions under its proposed optimal treatments are not or only slightly biased by hidden confounders. An example of a counterfactual matched patient pair is provided in Supplementary Fig. 4.

### OptAB’s selected optimal antibiotics exhibit faster efficacy compared to the administered antibiotics

**Fig. 5:**
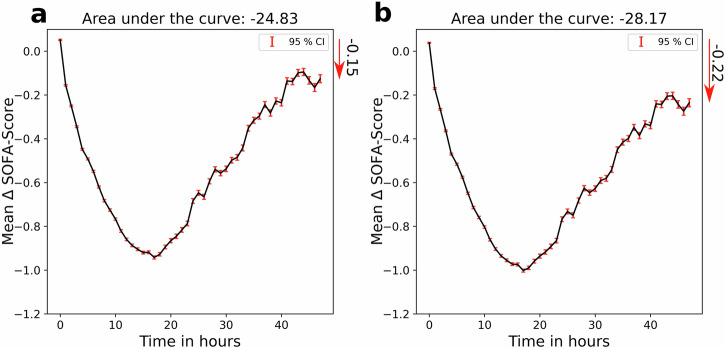
Estimated treatment effect for the optimal antibiotics compared to the factual treatment. The average difference of the SOFA-score for the optimal treatment according to OptAB's predictions relative to the factual treatment. The optimal treatment was defined as the antibiotic(s) combination leading to the lowest SOFA-score after the initial treatment phase of 48 h indicated by the red arrows. The bars indicate 95%-confidence intervals. In **a** the side-effects and contraindications were taken into account, whereas in **b** the treatment with the highest reduction of the SOFA-Score regardless of the side effects was selected. The area under the curve indicates the treatment effect integrated over time.

To estimate the average treatment improvement by OptAB we compared the SOFA-score time courses of each patient in the test set under the factual treatment with the optimal treatment predicted by OptAB. Here, we focus on the initial treatment phase of 48 h. The optimal treatment was defined as the antibiotic(s) leading to the lowest SOFA-Score after this initial 48 h phase. We considered two different scenarios (see Fig. 5): (a) Selection of the optimal treatment under the constraints imposed by side effects and contraindications and (b) minimal SOFA-score regardless of side effects. The mean reduction of the SOFA-score after 48 h is relatively low under both scenarios (−0.15 and −0.22). However, the efficacy of the optimal treatments is predicted to be more rapid with the highest SOFA-score reduction of ≈ 1 unit after 18 h.

Overall, the results indicate that an optimal selection of the initial antibiotic(s) could lead to a faster SOFA-score reduction and a slightly lower SOFA-score after the first treatment iteration at 48 h. Further plots including all iterations of the optimal treatment selection can be found in the Supplementary material (see Supplementary Fig. 8).

### OptABs optimal treatment selection to prevent antibiotic-induced side-effects

Current guidelines^35^ and recommendations^32–34^ indicate cases, where certain antibiotics are contraindicated. However, these recommendations can only be implemented on the basis of laboratory values and anamnesis at the moment of the treatment decision. Future side effects are sometimes difficult to anticipate. For example, in our test set we found that 125 (30.6 %) of the 409 patients who were factually treated with the nephrotoxic Vancomycin had acute kidney injury already at treatment initialization or developed acute kidney injury during the course of treatment. The high incidence of acute kidney injury is likely caused by high Vancomycin dosages. Patients in the MIMIC-IV dataset treated with Vancomycin typically receive 1 mg every 12 or 24 h, despite recommendations for dose control to reduce renal toxicities. Most dosing regimes aim to achieve Vancomycin serum levels of 20–30 μg/mL and depend on the patient’s weight or comorbidities, but they vary in terms of dose adjustment^39–41^.

OptAB identifies contraindications or liver-associated side-effects in 64 (19.1 %) of the 298 patients who received the hepatotoxic Ceftriaxone. Typically, either 1 g or 2 g of Ceftriaxone was administered once a day.

We checked, whether OptAB’s predictions could be helpful in preventing anticipated toxic side effects *before* a contraindicative event was detected. To this end, we selected all patients with a contraindication for the factual treatment they received during the disease course. For these patients, we counted, how often the optimal treatment under the side effect constraints suggested by OptAB excluded the contraindicated antibiotic in the optimal treatment.

OptAB’s optimal treatment excluded Vancomycin for 49 (39.2%) patients of the 125 patients who were factually treated with Vancomycin despite a kidney injury associated contraindication. As for hepatotoxicity, OptAB would have recommended an optimal treatment excluding the hepatoxic Ceftriaxone in 7 out of the 64 patients (10.9%) who received Ceftriaxone despite a liver injury associated contraindication.

These results indicate the potential of predictive models like OptAB to reduce antibiotic toxicity. In particular, OptAB can identify high-risk patients for Vancomycin-induced acute kidney injury and propose an alternative treatment. As an alternative to discontinuing treatment with Vancomycin, physicians can adjust Vancomycin dosages according to dosing recommendations. Vancomycin was administered to more than 50% of all Sepsis patients in the MIMIC-IV dataset possibly due to its sensitivity against MSSA although one of the main sources of sepsis are bacterial infections of the kidneys or the urinary system. However, a more individualized initial antibiotic selection should help to prevent kidney toxicity and to some extend liver toxicity.

### Antibiotic treatment decisions for individual patients

**Fig. 6:**
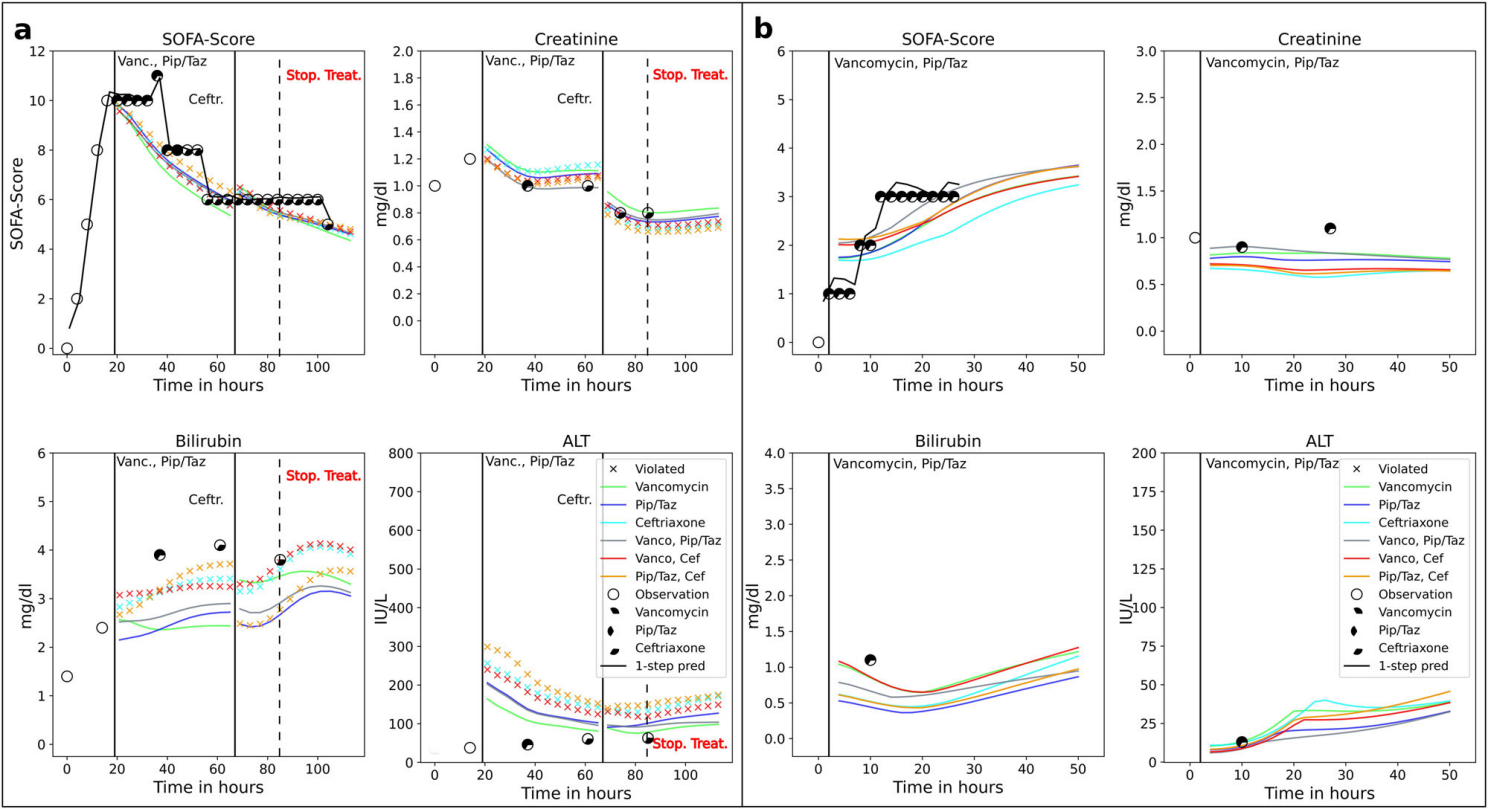
Individualized treatment effect predictions for two patients. OptAB's predictions of the SOFA-score and side effect associated laboratory values creatinine, bilirubin total and alanine transaminase for two different patients (**a**) and (**b**). The factual data for the patients are shown as black circles which are filled to indicate the antibiotics actually received by the patient (see legend). For the SOFA score we also show the one-hour forecasts as black lines for the factual treatment. The colored lines are the counterfactual long-term predictions for all pairwise combinations of the antibiotics Vancomycin, Piperacillin/Tazobactam and Ceftriaxone. The vertical black lines indicate the evaluation times of the treatment efficacy and the start of the next optimal antibiotic selection iteration based on further predictions of OptAB by assimilating all observed measurements up to that time. Crosses indicate that antibiotic-specific thresholds are violated for this treatment. The optimal treatment can be selected as the one with the long-term prediction (line) with the smallest SOFA-score in the treatment window at the next evaluation time point. Please note the different scales of the plots.

In Fig. 6, we compare observed data for two patients under actual treatment with counterfactual predictions based on alternative antibiotic regimens. For these predictions, we assume the initial treatment remains unchanged for at least 48 h, unless side-effect contraindications arise or new pathogen resistance results are obtained. As noted, antibiotic efficacy is typically assessed after 48 h and should not be adjusted beforehand^9,26,27^. Therefore, initial treatment decisions and adjustments at this point must rely on long-term forecasts up to 48 h ahead. Additionally, we present one-hour SOFA score forecasts for the actual treatment. The two patients were selected to illustrate how OptAB can improve antibiotic selection by suggesting alternative treatments that either reduce the SOFA score or minimize side effects, compared to the factual treatment. These cases highlight different scenarios where OptAB’s recommendations deviate from or align with the physician’s choice, demonstrating its potential to support individualized treatment. Further patient examples from the MIMIC-IV and AmsterdamUMCdb datasets are included in the Supplementary Material (see Supplementary Figs. 3 and 7).

#### Patient a

Patient *a* received the first treatment 19 h after sepsis onset with a SOFA-Score of 10 (see Fig. 6a). The initial choice of the clinician was a combination of Vancomycin and Piperacillin/Tazobactam. Then, 48 h after sepsis onset (29 h after administrating the first antibiotics), the treatment was changed to Ceftriaxone. In cases of positive pathogen detection, it is common practice to discontinue the administration of broad-spectrum antibiotics as vancomycin and piperacillin/tazobactam and switch to a more targeted therapy. However, no pathogens were found in this patient despite microbiological examination.

Over the complete time period, the one-hour forecasts of the SOFA-Score under the factual treatment are very accurate. The long-term forecasts under the factual treatment effectively capture the dynamics of the SOFA-Score and correctly predict the decrease from 10 at 19 h to 6 at 67 h after Sepsis onset (48 h after treatment initiation). However, OptAB’s long-term predictions can not anticipate the discrete jumps of the SOFA score. At the first evaluation time point 48 h after treatment initiation (67 h after sepsis onset), new predictions are initialized based on the assimilated data under the factual treatment up to this time point. This explains the jump in the long term predictions at 67 h after Sepsis onset (see Fig. 6a).

OptAB predicts correctly a decrease in creatinine for the combination of Vancomycin and Piperacillin/Tazobactam from 1.2 mg/dl to 1 mg/dl aligning with the two measured creatinine values of 1 mg/dl at 36 and 63 h after Sepsis onset. However, the predicted creatinine values do not differ much between the long-term predictions under the different factual and counterfactual treatments.

The predicted increase of bilirubin total from 2.5 mg/dl to 3 mg/dl is correct in its tendency for the factual treatment, but OptAB underestimates the observed bilirubin total values of 3.9 mg/dl at 37 h after Sepsis onset and 4.1 mg/dl at 63 h after Sepsis onset. The increase of bilirubin total is predicted to be higher for Ceftriaxone and its treatment combinations compared to treatments without Ceftriaxone after 48 h in agreement with the hepatotoxicity of Ceftriaxone. Individual predictions of the alanine transaminase are again more difficult, for the reasons described previously.

Based on the counterfactual predictions of OptAB, we would suggest Vancomycin as the initial antibiotic instead of the combination of Vancomycin and Piperacillin/Tazobactam and the following switch to Ceftriaxone. The lower bilirubin values combined with the predicted SOFA score suggest that a treatment with Vancomycin would have been similarly efficient whilst imposing a lower hepatotoxic risk to the patient.

#### Patient b

The treatment of example patient *b* with the combination of Vancomycin and Piperacillin/Tazobactam starts 3 h after Sepsis onset with a SOFA-Score of 1 increasing to 3 at 10 h after Sepsis onset (see Fig. 6b). The patient was discharged from the Intensive Care Unit 27 h after Sepsis onset probably due to a relatively stable health condition.

The one hour forecast of the SOFA-Score for the factual treatment is very accurate. The 48 h forecast for this factual treatment overestimates the SOFA-Score in the early treatment phase (<5 h), but the predicted increase to 3 at 25 h after Sepsis onset is also seen in the observed data. The prediction of a further increase to 3.6 at 48 h can not be evaluated, because the patient was released from the Intensive Care Unit after 27 h. For the counterfactual treatments, OptAB predicts the lowest SOFA-Score for Ceftriaxone over the entire 48 h after Sepsis onset.

The predictions of the side effect indicators creatinine, bilirubin total and alanine transaminase under the factual treatment with a combination of Vancomycin and Piperacillin/Tazobactam are difficult to validate, because of the small number of measurements. The observed value for creatinine 1.2 mg/dl at 27 h after Sepsis onset and the bilirubin total measurement of 1.2 mg/dl at 10 h after Sepsis onset are underestimated by OptAB. However, all the indicators for contraindications are safely within their constraints. Based on the projection that the SOFA-score is consistently the lowest for Ceftriaxone and contraindications are unlikely, OptAB would suggest a treatement with Ceftriaxone for patient *b*.

## Discussion

In this work we presented the Artificial Intelligence model OptAB, which is to the best of our knowledge the first data-driven and online-updateable optimal antibiotic selection model for Sepsis accounting for side-effects and contraindications. OptAB extends the Treatment-Effect Controlled Differential Equations^25^ and can handle irregular measurements, a large proportion of missing values and time-dependent confounding.

We demonstrated that OptAB can predict both the SOFA score as an important indicator for the Sepsis disease state as well as side effects indicators creatinine, bilirubin total and alanine transaminase under treatment combinations of the antibiotics Vancomycin, Ceftriaxone and Piperacillin/Tazobactam. We provide evidence, that OptAB captures the toxic side effects and contraindications, which are important for treatment decisions. We have also illustrated how dynamic models like OptAB can be used to optimize the selection of the best antibiotic treatment for an individual patient, based on its characteristics. Such dynamic treatment regimes can potentially lead to better Sepsis-outcomes, shorter ICU stays and less side effects.

One common issue with machine learning models like OptAB is performance decline when applied to new environments with different data distributions^42^. To address this, we tested OptAB, trained on MIMIC-IV, on the AmsterdamUMCdb dataset^36^, which includes 6285 sepsis patients (364 treated with Ceftriaxone, 35 with Vancomycin, and none with Piperacillin/Tazobactam). Despite differences across datasets from two continents, OptAB still predicted SOFA scores and key side effects (creatinine, total bilirubin, and alanine transaminase). Although SOFA-Score and creatinine predictions were less accurate with shorter prediction horizons, total bilirubin and alanine transaminase predictions improved (see Supplemental material), likely due to more frequent data collection in AmsterdamUMCdb. These results highlight OptAB’s ability to capture critical sepsis characteristics and the importance of high-quality patient data. Further research is needed to address distribution shifts in dynamic models like OptAB.

An important limitation of our work is that counterfactual predictions cannot be tested in reality, as each patient receives only one factual treatment. Previous simulations^25^ support our assumption that the TE-CDE modeling approach provides accurate counterfactual outcome predictions when known confounders are present. However, unknown confounders in observational data may lead to biased treatment effects. Despite this, OptAB’s promising results on unseen external test data and its ability to learn realistic treatment effects related to known antibiotic toxicities suggest that unknown confounders do not drastically bias OptAB’s predictions of disease progression. Additionally, we have shown that OptAB’s predictions of optimal counterfactual treatments align with the disease progressions of similar patients actually treated with the suggested optimal regimen, as indicated by a comparison of mean absolute errors (see Fig. 4 and Supplementary Material Fig. 4). Furthermore, we are exploring how to make these dynamic predictions explainable to enhance medical insights into sepsis and improve trust in decision support systems based on AI models like OptAB. A prospective clinical study will be crucial to evaluate OptAB’s performance and the clinical relevance of its predictions in real-world scenarios, providing valuable insights into their reliability in practice.

While bacterial infections account for 80–90% of all sepsis cases, approximately 10–20% are due to fungal pathogens^5,6^. Fungal infections often arise as secondary infections following bacterial or viral sepsis. In co-infections with both bacterial and fungal pathogens, treatment must include both antimicrobials and antifungals, such as Amphotericin B^43^. Viral sepsis is relatively rare, except for SARS-CoV-2, influenza, and RSV. During the COVID-19 pandemic, the incidence of SARS-CoV-2-driven sepsis exceeded 15%, though it has since declined, as the omicron variant rarely leads to sepsis^7,44,45^. Distinguishing between pathogens and non-pathogens is challenging, as the interpretation of microbiological findings depends on factors like patient history, individual characteristics, and the location of the culture sample. When a potential fungal or viral pathogen is detected, OptAB notifies the attending physician and suggests an optimal antibiotic regimen, but the final decision on antibiotic administration rests with the physician.

Our selection of antibiotics was primarily based on data availability and sepsis treatment guidelines. While we focused on three antibiotics, which represent a small subset of those available for sepsis, the Surviving Sepsis Campaign recommends tailoring antibiotic choices to local patterns of resistant pathogens^9^. Consequently, our data-driven selection reflects local treatment protocols based on regional pathogen prevalence. Future versions of OptAB should incorporate epidemiological features to address regional variations in bacterial pathogens, which significantly influence treatment decisions^46,47^.

Future research will focus on uncertainty quantification for OptAB’s predictions and managing time-varying confounding in continuous treatments, allowing for dose optimization. In the MIMIC-IV dataset, antibiotics were often administered according to standard regimens, despite guidelines suggesting personalized dose adjustments, particularly for Vancomycin^39–41^. As a result, we could not perform dose optimization. Most dosing regimens aim for specific serum levels and depend on patient factors like weight and comorbidities. Extending OptAB to optimize dosing would allow it to adjust dosages based on individual side-effect risks, such as reducing Vancomycin dosages for patients at high risk of acute kidney injury.

While the appropriate use of vasopressors and fluid balancing affects sepsis outcomes, there are no significant interactions between these treatments and antibiotic therapy^48–50^. Therefore, optimal antibiotic recommendations are independent of vasopressor and fluid management strategies. However, some antibiotics, like macrolides and fluoroquinolones, may interact with non-antibiotic treatments, including statins and antacids^51,52^. Future work could enhance OptAB to account for such interactions, provided sufficient data on non-antibiotic treatments are available.

The primary goal of sepsis treatment is to prevent death. We selected the SOFA score as the endpoint for OptAB rather than mortality because it provides detailed information about the patient’s disease state, while mortality is a binary outcome. A high SOFA score is strongly associated with increased mortality, so OptAB implicitly aims to reduce mortality by lowering the SOFA score^53–55^. Current sepsis treatment guidelines recommend initiating antibiotics within one hour after sepsis onset, regardless of mortality risk^9^. Thus, the SOFA score may be a more suitable marker for the initial choice of antibiotics. Future work could explore developing a hybrid model that combines OptAB with survival analysis to predict individual survival probabilities, alongside the SOFA score and side-effect-related laboratory values.

Another avenue for future research is applying OptAB to patients in general wards. While the SOFA score is derived from ICU data, early detection scores for sepsis in non-ICU settings, such as the quick SOFA score or NEWS2 (National Early Warning Score 2)^56,57^, exist, they have limited prediction accuracy^58,59^. With sufficient data quality and quantity, future versions of OptAB could incorporate predictions of procalcitonin levels as an alternative marker for assessing antibiotic treatment efficacy, although procalcitonin levels are not measured in MIMIC-IV.

## Methods

In this section we provide an overview of data preprocessing, introduce the TE-CDE^25^ and finally describe OptAB in detail.

### Optimal antibiotic selection by OptAB

OptAB iteratively selects optimal antibiotic treatments by minimizing the SOFA score associated with treatment success while considering drug-related side effects. A reliable assessment of initial treatment efficacy occurs 48 h after administration, during which the treatment should remain unchanged, except for results from microbiological cultures, resistance tests, and laboratory values indicating severe contraindications. After the initial evaluation and any necessary adjustments, efficacy should be reassessed every 24 h^9,26,27^.

Given the life-threatening nature of sepsis, the primary treatment goal is to combat the infection, thereby reducing the SOFA score. Antibiotic administration should be avoided if contraindicated, and among equally effective treatments, the one with the least side effects should be preferred. Consequently, OptAB iteratively selects the antibiotic (or combination of antibiotics) that minimizes the SOFA score after 48 or 24 h while adhering to specific thresholds for contraindications and side effects.

At each iteration all available measurements are fed into OptAB to obtain updated predictions. OptAB excludes antibiotics with known resistance against pathogens from its treatment optimization. Once a side-effect associated threshold is violated, OptAB raises warnings to identify high-risk patients for antibiotic-associated side-effects and propose alternative treatment options. If the SOFA-Score is decreasing over a period of 48 h, OptAB recommends de-escalating antibiotic treatment according to common guidelines for Sepsis and antibiotic de-escalation^9,26^.

OptAB provides both an optimal treatment recommendation and predictions of the SOFA-Score and laboratory values indicating contraindications and side-effects for all potential treatments. This provide physicians a more comprehensive assessment of possible treatment options for individual patients.

OptAB is based on the Treatment-Effect Controlled Differential equation (TE-CDE) approach^25^ and accounts for time-dependent confounding. We extended the TE-CDE to optimize dynamic treatment regimes under a realistic medical setting using real-world patient data.

The original TE-CDE comprises an encoder and a decoder, both modeled as Neural CDEs^60^. The encoder is trained for short-term forecasts using all observed covariates up to time point *t* and computes an informative latent disease state, which serves as the initial value for the decoder trained for long-term forecasts. To ensure a balanced representation of the latent disease state^61^, we incorporate a penalty term for the prediction accuracy of future treatments based on this latent state^25^. This approach mitigates the time-dependent confounding effects between the latent disease state and future treatments.

**Fig. 7:**
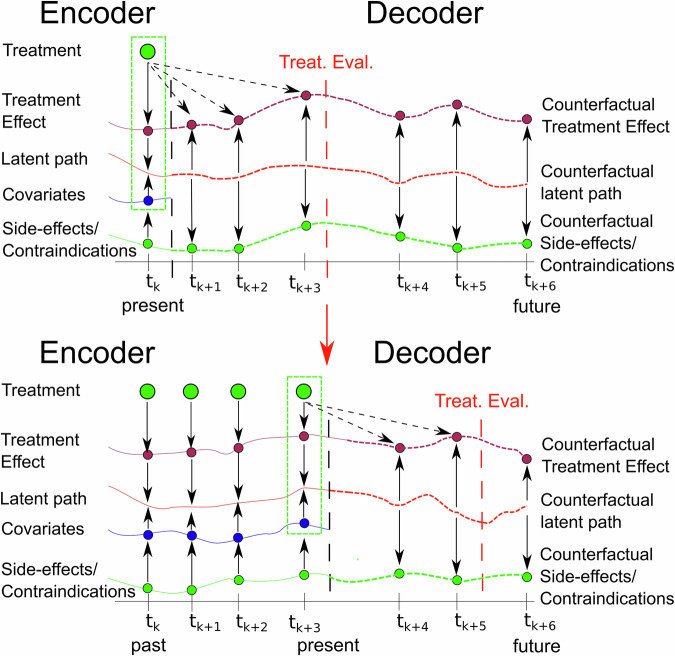
Methodological outline of the online-updateable optimal treatment selection model OptAB. OptAB is based on a TE-CDE^25^ model consisting of an encoder and a decoder. The encoder assimilates all observed data of a patient until the present time point to create a latent disease state which is used by the decoder as an initial value for long-term forecasts. By comparing the predicted disease courses under different antibiotic treatments, the most efficient treatment with tolerable side effects can be selected.

During inference, the encoder assimilates the available data for an individual patient from the past to the present and generates the patient’s latent disease state to initialize the trained decoder. The decoder then forecasts the future disease course, including the SOFA score and laboratory values indicating side effects, under the specified treatment. When new data arrives or the treatment changes, the encoder updates the latent disease state, which subsequently refreshes the decoder’s long-term forecasts (see Fig. 7).

### Datasets

We trained and tested OptAB on the large real-world EHR dataset MIMIC-IV^29,30^ containing over 450.000 admissions to the Beth Israel Deaconess Medical Center of the Harvard medical School including demographics, laboratory values, vital values, medications and diagnosis data. We tested OptAB on AmsterdamUMCdb containing 23.106 admissions from 2003 to 2016 to the intensive care unit of the Amsterdam University Medical center^36^.

### Legal and ethical considerations

For Mimic-IV, the collection of patient information and creation of the research was reviewed by the Institutional Review Board at the Beth Israel Deaconess Medical Center, who granted a waiver of informed consent and approved the data sharing initiative^29,30^.

For AmsterdamUMCdb, an internal team carried out a data privacy impact assessment and an external team performed an audit. The external team was led by a member of the privacy expert group at the Netherlands Federation of UMCs. The Regional Medical Ethics Committee confirmed that the creation of AmsterdamUMCdb was not eligible for their assessment as no specific research question was involved. The Ethics in Intensive Care Medicine group provided external ethics review and appraisal^36^.

Both datasets were shared by the data owners for research purposes upon request and under the supervision of a practicing intensive care physician (Dr. Ingobert Wenningmann) in AmsterdamUMCdb. No further ethical approval was required as no further data was used.

### Definition of Sepsis

For SOFA-Score calculation and Sepsis-3 classification adhering to the latest Sepsis-3 criteria^56,62^ we employed the OpenSep pipeline^63^ for the MIMIC-IV dataset and the ricu R package for AmsterdamUMCdb^64^. Patients with a SOFA-Score greater than or equal to 2 occurred within 48 h before or 24 h after a suspicion of infection (SOI) were classified as Sepsis patients. A suspicion of infection is defined as either the administration of antibiotics followed by sampling of bodily fluids for microbiological cultures within 24 h or sampling of bodily fluids for microbiological cultures followed by the administration of antibiotics within 72 h. The onset of Sepsis is the earlier event of sampling for microbiological cultures or the administration of antibiotics.

### Data preprocessing

We focussed on patients admitted to the Intensive Care Unit since the SOFA-Score can only be determined using data collected at the ICU and extract all patients treated with Vancomycin, Piperacillin/Tazobactam and Ceftriaxone. To avoid bias from treatment effects of other antibiotics we excluded all patients who additionally received other antibiotics. We modeled the three antibiotics as binary features, because they were typically administered according to standard treatment regimes in our datasets. This enables OptAB to predict the (long-term) effects of antibiotic treatments rather than the (short-term) effects of individual administrations.

Before training of OptAB we performed a variable selection considering all observed laboratory variables and vital parameters as potential time-dependent predictors. We excluded all variables measured in less than 50% of the patients. Then, we calculated the absolute Spearman-correlation between the SOFA-Score and the potential covariables. Due to sparse and highly irregular measurements of the laboratory values and vital signs, we divided the data into 12 h intervals for the calculation of the Spearman-correlation. We selected all variables with an absolute Spearman-correlation of more than 0.3 in at least one time interval. Plots of the Spearman-correlation can be found in our Github repository (https://github.com/philippwendland/OptAB).

The selected time-dependent features are the SOFA-Score, alanine transaminase (in IU/L), anion gap (in mEq/L), bicarbonate (in mEq/L), bilirubin total (in mg/dl), blood urea nitrogen (in mg/dl), creatinine (in mg/dl), diastolic blood pressure (in mmHg), number of platelets (in k/uL), red cell distribution width (in %) and systolic blood pressure (in mmHg). The selected static features are biological sex, age, height and weight at submission.

We included cumulative missing masks as extra variables as the missingness pattern in Electronic Health Records often contains important information about the patient’s state^65^. These masks describe whether a variable is observed or not^60^.

### Mathematical notation

In this subsection we introduce some basic notations. For each patient *i*, let $Z=({z}_{{t}_{0}},{z}_{{t}_{1}},...,{z}_{{t}_{K}})\in {{\mathbb{R}}}^{L\times K}$ be a matrix of the temporal covariates with *t*_0_ corresponding to the latest of Sepsis onset or first measured SOFA-Score. *Z* contains missing values, because the variables are usually measured at different frequencies and times. Let $A=({a}_{{t}_{0}},{a}_{{t}_{1}},...,{a}_{{t}_{K}})\in {{\mathbb{R}}}^{M\times K}$ be a matrix of the antibiotic treatments with ${a}_{{t}_{j}}^{m}=1$ if the patient is treated at *t*_*j*_ with antibiotic *m* and ${a}_{{t}_{j}}^{m}=0$ otherwise. In our context, treated at *t*_*j*_ implies that the treatment is ongoing. Let $d\in {{\mathbb{R}}}^{D}$ be the static variables. *Y* ⊂ *Z* with $Y=({y}_{{t}_{0}},{y}_{{t}_{1}},...,{y}_{{t}_{n}})\in {{\mathbb{R}}}^{R\times K}$ is the outcome corresponding to the patient’s disease state and side-effects indicating laboratory values.

### Neural differential equations

Neural Ordinary Differential Equations (Neural ODEs)^66^ are a hybrid neural network approach modeling the right-hand side of an ODE as a neural network. Let ${f}_{\phi }:{{\mathbb{R}}}^{P}\to {{\mathbb{R}}}^{P}$ and ${g}_{\varphi }:{{\mathbb{R}}}^{L}\to {{\mathbb{R}}}^{P}$ be neural networks and let ${z}_{{t}_{0}}\in {{\mathbb{R}}}^{L}$ be a vector of covariables measured at *t*_0_. A Neural ODE can be defined as

1 $${x}_{{t}_{0}}={g}_{\varphi }\left({z}_{{t}_{0}}\right),{x}_{t}={x}_{{t}_{0}}+{\int_{{t}_{0}}^{t}}{f}_{\phi }\left({x}_{s}\right)ds$$

The predictions of standard Neural ODEs are based solely on data measured at *t*_0_ and do not incorporate subsequent measurements.

Neural Controlled Differential Equations^60^ extend Neural ODEs by solving a Riemann-Stieltjes-Integral instead of a classic Riemann-Integral to process subsequent measurements, irregularities and missing values of time series data. Let ${F}_{\theta }:{{\mathbb{R}}}^{P}\to {{\mathbb{R}}}^{P\times \left(L+1\right)}$ and ${g}_{\eta }:{{\mathbb{R}}}^{L}\to {{\mathbb{R}}}^{P}$ be neural networks depending on parameters *θ* and *η*. Let ${{\mathcal{X}}}_{Z}:\left[{t}_{0},t\right]\to {{\mathbb{R}}}^{\left(L+1\right)}$ be an interpolation of a time series $Z=({z}_{{t}_{0}},...,{z}_{{t}_{K}})\in {{\mathbb{R}}}^{L\times K}$ such that ${{\mathcal{X}}}_{Z}({t}_{j})=({t}_{j},{z}_{{t}_{j}})$. A Neural CDE is defined as the solution $x\left(t\right)$ to

2 $${x}_{{t}_{0}}={g}_{\eta }\left({z}_{{t}_{0}}\right),{x}_{t}={x}_{{t}_{0}}+{\int_{{t}_{0}}^{t}}{F}_{\theta }\left({x}_{s}\right)\frac{d{{\mathcal{X}}}_{Z}\,\left(s\right)}{ds}ds$$

Often ${{\mathcal{X}}}_{Z}$ is called driver or control of the Neural CDE. To ensure causality and online-updateability we use the rectilinear interpolation scheme^67^.

### Treatment-effect controlled differential equation (for predicting the disease state of Sepsis patients and antibiotic-associated side-effects)

The TE-CDE^25,60^ consists of an Encoder and decoder both modeled as Neural Controlled Differential equations. Here we describe the extensions to antibiotic treatment optimization taking side-effects into account (see Fig. 7).

The Encoder is trained for short-term predictions until *t* + *ϵ* ≥ *t*_0_, where *ϵ* corresponds to the forecast horizon. The Encoder assimilates all variables *Z* and treatments *A* measured up to the current timepoint *t*.

3 $${x}_{{t}_{0}}={g}_{\eta }\left({z}_{{t}_{0}},{a}_{{t}_{0}},d\right),{x}_{t+\epsilon }={x}_{{t}_{0}}+{\int_{{t}_{0}}^{t+\epsilon }}{F}_{\theta }\left({x}_{s}\right)\frac{d{{\mathcal{X}}}_{Z,A}\left(s\right)}{ds}ds$$

The decoder is trained for long-term predictions up to ${t}^{{\prime} }\, >\, t+\epsilon$ and is initialized by a transformation of the latent disease state of the Encoder *x*_*t*+*ϵ*_ at *t* + *ϵ* and the static variables *d*. We set *ϵ* = 1*h* for the disease prediction of Sepsis patients

4 $${x}_{{t}^{{\prime} }}={g}_{\varphi }\left({x}_{t+\epsilon },d\right)+{\int_{t+\epsilon }^{{t}^{{\prime} }}}{f}_{\phi }\left({x}_{s}\right)ds$$

Although it is possible to drive the decoder by a future treatment scheme *A* it is not reasonable in our use case, because the efficacy of initial antibiotics can only be evaluated 48 h after treatment and should be re-evaluated every 24 h^9,26,27^. Therefore, we set the long-term prediction horizon to ${t}^{{\prime} }=t+48h$ or ${t}^{{\prime} }=t+24h$. The decoder requires no control *A* and is therefore simplified to a standard Neural ODE. If an antibiotic needs to be changed due to contraindications or its efficacy is evaluated at *t*_change_, further observations of the covariables are available. These observations are fed into the encoder to derive an updated latent disease state at *t*_change_ + *ϵ*, which is then used to initialize the decoder.

We use two neural networks ${h}_{\alpha }:{{\mathbb{R}}}^{P}\to {{\mathbb{R}}}^{R}$ and ${h}_{\beta }:{{\mathbb{R}}}^{P}\to {{\mathbb{R}}}^{M}$ to calculate the forecasts of the outputs ${\hat{y}}_{s}={h}_{\alpha }\left({x}_{s}\right)$ and treatment probabilities ${\hat{p}}_{s}=\sigma ({h}_{\beta }\left({x}_{s}\right))$ where *σ* corresponds to the Softmax activation function.

### Time-dependent confounding

One major challenge for estimating counterfactual outcomes with longitudinal observational data is the influence of time-dependent confounders on treatment administration^24,68^. The presence of confounders can lead to biased causal effect estimations. The Treatment-Effect Controlled Differential Equation penalizes the prediction accuracy of the treatment to obtain a balanced representation of the latent disease state. This counteracts time-dependent confounding^25,61,69^. Concretely, the Binary Cross Entropy of the treatment prediction is maximized to ensure, that the latent representation *x* is not predictive of the future treatment administration. Of course, the latent representation is influenced by past treatments.

### Training and objective function of the TE-CDE^25^

First, we trained the encoder to obtain a reliable initialization for the decoder $x\left(t+\epsilon \right)$. The decoder was trained afterwards. The loss function for the encoder and the decoder consists of the MSE of the outcome prediction ${{\mathcal{L}}}_{y}=\frac{1}{K-{t}_{{\rm{start}}}}\mathop{\sum }\nolimits_{j = {t}_{{\rm{start}}}}^{K}\mathop{\sum }\nolimits_{r = 0}^{R}{({y}_{{t}_{j}}^{r}-{\hat{y}}_{{t}_{j}}^{r})}^{2}$ and the Binary Cross Entropy loss of the treatment prediction ${{\mathcal{L}}}_{a}=\frac{1}{K-{t}_{{\rm{start}}}}\mathop{\sum }\nolimits_{j = {t}_{{\rm{start}}}}^{K}\mathop{\sum }\nolimits_{m = 0}^{M}{a}_{{t}_{j}}^{m}\log ({\hat{p}}_{{t}_{j}}^{m})+(1-{a}_{{t}_{j}}^{m})\log (1-{\hat{p}}_{{t}_{j}}^{m})$. The loss function for the encoder and the decoder is defined as

5 $${\mathcal{L}}=\frac{1}{n}\mathop{\sum }\limits_{i}^{n}{{\mathcal{L}}}_{y}^{i}+\mu {{\mathcal{L}}}_{a}^{i}$$

We set *t*_*start*_ = 1 for the training of the Encoder. For the decoder *t*_*start*_ varies between 2 and 119 corresponding to the last time when more than 10 % of the patients are located at the Intensive Care Unit. The parameter *μ* controls the trade-off between outcome prediction and adjustment for treatment confounding.

### Study design and selection of antibiotics

For the optimal antibiotic selection we focus on the three antibiotics Vancomycin, Piperacillin/Tazobactam and Ceftriaxone. The selection of these three antibiotics is based on both medical knowledge and data availability. First, we selected the most frequently administered antibiotics, specifically those administered to at least 5000 among the 26.111 hospitalizations with Sepsis in the MIMIC-IV dataset (see Table 1).

**Table 1:** Descriptive statistics for all antibiotics administered to more than 5000 Sepsis patients in the MIMIC-IV dataset

Antibiotics	Patients treated with	Only treated with	90-day death rate
All patients	26.111		30.1 %
Vancomycin	14.796 (56.7 %)	1.021	40.7%
Cefepime	6.945 (26.6 %)	133	50.3%
Piperacillin/Tazobactam	5.938 (22.7 %)	280	46.4%
Cefazolin	5.687 (21.8 %)	2.961	13.8%
Ceftriaxone	5.389 (20.6 %)	1.144	35.6%

Table presenting for each antibiotic administered to more than 5000 patients in the MIMIC-IV dataset the count of patients (only) treated with that specific antibiotic and the corresponding 90-day death rate.

More than 50% (14,795) of all Sepsis patients were treated with Vancomycin. Vancomycin is one of the first-line antibiotics for the treatment of Sepsis and is effective against a broad spectrum of gram-positive pathogens including MRSA^31^. Unfortunately, Vancomycin is renal toxic and causes many acute kidney injuries^32,35^. Consequently, the administration of Vancomycin to patients with an impaired kidney function or acute kidney injury should be avoided whenever possible.

Piperacillin/Tazobactam was administered to 5937 (22.7%) and Ceftriaxone to 5389 (20.6%) of the Sepsis patients. Both are commonly recommended for Sepsis treatment^9^. Unfortunately, Ceftriaxone is hepatotoxic and can cause liver injuries, especially in patients with comprised livers or liver impairments^33,34^.

Cefepime was administered to 6944 patients and Cefazolin was administered to 5687 patients. Patients treated with Cefazolin had a much lower 90-day mortality rate, indicating that some of the patients might not have suffered from severe Sepsis or that Cefazoline was administered for other purposes, like e.g. cellulitis or other skin infections. Only 133 patients were exclusively treated with Cefepime, which is not sufficient for reliably estimating treatment effects. Therefore, we excluded these two antibiotics from our model.

### Identification of contraindications and patients at high-risk for side-effects

We defined thresholds for laboratory values indicating patients at high risk for side-effects and contraindications. Due to Vancomycin’s nephrotoxicity we recommend avoiding treatment with Vancomycin in patients with (at least) stage-1 acute kidney injury or a creatinine value exceeding 2 mg/dl. According to current guidelines^35^ Stage-1 Acute Kidney Injury was defined as either an increase in creatinine of 0.3 mg/dl within 48 h, or an increase of 1.5 times the baseline creatinine within the previous 7 days or an urine output of less than 0.5 ml/kg/h for 6–12 h. We include the creatinine value itself as a contraindication, because Vancomycin administration should be avoided not only in cases of acute kidney injury, but also in patients with chronic kidney injuries^70^.

Due to the absence of thresholds indicating hepatotoxic contraindications of Ceftriaxone, OptAB relies on thresholds for discontinuing hepatotoxic drugs in tuberculosis treatment. Treatment with hepatotoxic drugs should be discontinued if the bilirubin total level exceeds twice the upper range (2.4 mg/dl for men and 2.2 mg/dl for women) indicating jaundice and bile duct inflammation, or if the alanine transaminase level exceeds five times the upper range (280 U/L) indicating toxic liver injuries and liver cell damages^71,72^. These thresholds can be adjusted to meet current guidelines or individual assessments by the physician.

Additionally, we compiled a list of sepsis-associated bacterial pathogens in MIMIC-IV that influence the antibiotic selection of OptAB, ensuring that non-pathogens have no influence on the antibiotic selection process. We focus on samples from abscesses, blood cultures, bronchial washings, bronchoalveolar lavage, sputum, tissue and urine as these are highly associated with Sepsis. The availability of samples from other bodily fluids is limited in MIMIC-IV or their association with Sepsis is negligible. Moreover, we compiled a list of potential fungal and viral pathogens in MIMIC-IV that are frequently associated with Sepsis (see https://github.com/philippwendland/OptAB). Unfortunately, AmsterdamUMCdb does not include microbiological data.

### Side effect comparison in Fig. 3

In Fig. 3 we accounted for the different number of patients factually treated with different antibiotics by downsampling. Individual patient characteristics can influence the treatment decision of physicians in observational data. This influence can lead to different characteristics among the patient groups factually treated with different antibiotics. In combination with different numbers of patients receiving each antibiotic (Vancomycin: 241, Ceftriaxone: 199 and Piperacillin/Tazobactam: 58) this could introduce bias. Therefore, we excluded patients treated with multiple antibiotics and then sampled from each of the factual treatment groups with equal probability without replacement. For these sampled patients we used OpAB to simulate treatment with Vancomycin and Ceftriaxone.

### Implementation details

We performed a train-, test- and validation split (80%, 10%, 10%) for hyperparameter optimization of OptAB including early stopping. Due to the large training time and memory usage of OptAB, we used a train-test split for the hyperparameter optimization instead of a five-fold cross validation. Training was performed using the PyTorch implementation of Adam and hyperparameter optimization was performed using the Optuna implementation of the Tree-structured Parzen Estimator (Tree-Parzen).

We normalized all variables ${\tilde{z}}_{i,l,{t}_{j}}=\frac{{z}_{i,l,{t}_{j}}-{\rm{mean}}\left({z}_{i,l,{t}_{j}}\right)}{{\rm{std}}\left({z}_{i,l,{t}_{j}}\right)}$, where ${\rm{mean}}\left({z}_{i,l,{t}_{j}}\right)$ and ${\rm{std}}\left({z}_{i,l,{t}_{j}}\right)$ represent the mean and standard deviation for variable *l* over all patients *i* and times *t*_*j*_.

Although OptAB can handle missing values over time, OptAB is not able to impute missing values at initialization. Consequently, we performed a K-Nearest-Neighbors (KNN) missing value imputation for the initial values of the covariates^73^.

Due to the usage of the computationally expensive rectilinear interpolation scheme of the Neural CDEs^60^ we rounded the timepoints of the covariates to hours, because otherwise we were not able to train and test OptAB as the control matrix becomes very large leading to a massive increase in training time and memory usage.

## Supplementary information

Supplementary Material

## Data Availability

The MIMIC-IV dataset^29,30^ is publicly available from physionet under https://physionet.org/content/mimiciv/2.2/. The data is shared by the data owners after reasonable request. AmsterdamUMCdb^36^ is available under https://amsterdammedicaldatascience.nl/amsterdamumcdb/. The data is shared by the data owners after reasonable request including the supervision of a practising intensivist.

## Code availability

The underlying code of OptAB, the data preprocessing and our experiments is available at https://github.com/philippwendland/OptAB.
