# Supplementary material for: An optimal antibiotic selection framework for Sepsis patients using Artificial Intelligence
Source: NPJ Digit Med. 2024 Nov 29;7:343. doi: 10.1038/s41746-024-01350-y (PMC11607445; doi:10.1038/s41746-024-01350-y)
Supplement: Supplementary file 1 — Supplementary Material [file 41746_2024_1350_MOESM1_ESM.pdf]

Supplementary Material to an optimal antibiotic selection framework for Sepsis patients using Artificial Intelligence

Philipp Wendland[1], Christof Schenkel-Häger[2], Ingobert Wenningmann[3], and Maik Kschischo[1,4,*]

[1]University of Applied Sciences Koblenz, Department of Mathematics and Technology, Remagen, 53424, Germany
[2]University of Applied Sciences Koblenz, Department of Economics and Social Studies, Remagen, 53424, Germany
[3]University Hospital Bonn, Department of Anesthesieology and Operative Intensive Care Medicine, Bonn, 53127, Germany
[4]University of Koblenz, Department of Computer Science, Koblenz, 56070, Germany
[*]kschischo@uni-koblenz.de

External validation using the AmsterdamUMCdb dataset

As an external validation we tested, how the OptAB model trained on the MIMIC-IV dataset performs on AmsterdamUMCdb [1]. AmsterdamUMCdb contains EHR data from 23.106 admissions to the intensive care unit of the Amsterdam University Medical center spanning from 2003 to 2016. The distribution of administered antibiotics in AmsterdamUMCdb differs from that in the MIMIC-IV dataset possibly due to different pathogens or local treatment protocols. Among the 6.285 sepsis patients 3.374 were treated with Ceftriaxone, 3.058 were treated with Cefotaxim and 1.718 were treated with Erythromycin.

However, for the external validation of our OptAB model trained on MIMIC-IV we focus on Sepsis patients exclusively treated with Vancomycin, Ceftriaxone and Piperacillin/Tazobactam. In the external test set, 364 patients were treated with Ceftriaxone, 35 patients were treated with Vancomycin and none was treated with Piperacillin/Tazobactam.

Similar to the MIMIC-IV dataset, OptAB forecasts the SOFA-Score at Sepsis onset for the next hour with a MSE of less than 25 %. The SOFA-Score forecasts for longer time windows are less accurate (see Supplementary Figure 1). Assimilating further data enables OptAB to forecast the SOFA-Score for the next hour with MSEs $<$ 2-4 %. Observing the patient for at least two hours after Sepsis onset enables OptAB to forecast the SOFA-Score with MSEs $<$ 15 % for 5-10 hours, instead of 10-15 hours as in the MIMIC-IV dataset. We observed that OptAB underestimates the SOFA-Score in AmsterdamUMCdb probably due a distribution shift of the SOFA score. The mean SOFA-Score over all Sepsis patients in AmsterdamUMCdb is 6.8, whereas the mean SOFA-Score over all Sepsis patients in MIMIC-IV is 4.43. OptAB forecasts of Bilirubin total are more accurate with MSEs $<$ 20 % for a very long forecast horizon. As in the MIMIC-IV dataset assimilating data for at least four hours after sepsis onset substantially improves the accuracy of creatinine forecasts. However, the forecast horizon for MSEs of 15-30 % is limited to 20 hours after assimilating data for 15 hours after Sepsis onset. The MSEs of alanine transaminase are often lower than 40 % for forecasts of the next 5 hours, but often exceed 80 % between 25 to 35 hours after Sepsis onset, before dropping to MSEs $<$ 30 % again. Despite being trained on the MIMIC-IV dataset, OptAB predicts bilirubin total and alanine transaminase with higher accuracy on the unseen external AmsterdamUMCdb cohort. This is likely due to improved data availability. In the AmsterdamUMCdb testset, 23,3 % of the patients had no recorded bilirubin total values and 16.8 % had no alanine transaminase recordings. In contrast, the MIMIC-IV testset had more missing data, with 56.8 % of patients without any bilirubin total recordings and 56.1 % without any alanine transaminase recordings.

In the same way as for the MIMIC-IV dataset, we compared the time courses of the side effect indicating laboratory values in response to treatment with Vancomycin and Ceftriaxone for each patient (see Supplementary Figure 2). Because no patient was treated with Piperacillin/Tazobactam and only 35 patients with Vancomycin, we downsampled the patients treated with Ceftriaxone to 35. After an initial transient, the densities of the differences of the labora-

tory values between the two time courses under Vancomycin versus Ceftriaxone treatment show the expected tendencies. Patients counterfactually treated with Vancomycin tend to have higher creatinine levels and lower bilirubin total and alanine transaminase levels after some hours of treatment compared to those counterfactually treated with Ceftriaxone.

To assess OptAB's ability to forecast the sepsis disease course and improve treatment decisions for individual patients, we present two test patients from AmsterdamUMCdb (see Supplementary Figure 3). The one-hour SOFA-Score forecasts are highly accurate for both patients. Although OptAB underestimates the SOFA-Score of 5 by 1 for patient a at 48 hours after treatment, OptAB correctly forecasts the decreasing trend in the SOFA-Score under the factual Ceftriaxone treatment. OptAB accurately forecasts the creatinine values of 0.5 mg/dl at 36 hours and 61 hours after Sepsis onset, but slightly overestimates the creatinine value of 0.4 at 85 hours after Sepsis onset. Due to a lower SOFA score forecast of approximately 0.5 under Vancomycin treatment 48 hours after treatment initialization, along with low creatinine levels and a predicted increase in bilirubin total and alanine transaminase for the factual treatment with Ceftriaxone, OptAB recommends Vancomycin for the treatment of patient a. OptAB correctly forecasts the decreasing trend of the SOFA-Score from 6 to 5 at 48 hours after treatment initialization under the factual Ceftriaxone treatment. OptAB accurately forecastss the creatinine levels of 4.1 mg/dl at 20 hours after Sepsis onset and 3.9 mg/dl at 28 hours after Sepsis onset, but slightly overestimates the creatinine level of 3 mg/dl at 52 hours after Sepsis onset under Ceftriaxone treatment. OptAB overestimates the bilirubin total levels shortly after treatment initialization. OptAB accurately forecastss the alanine transaminase level of 155 IU/L at 48 hours after treatment start. Due to elevated creatinine levels of patient b and similar SOFA-Score forecastss for all treatments, OptAB recommends to treat patient b with Ceftriaxone or Piperacillin/Tazobactam.

All in all, OptAB's performance only slightly decreases when transferred to AmsterdamUMCdb, but OptAB tends to underestimate the SOFA-score probably due to a distribution shift of the SOFA-Score.

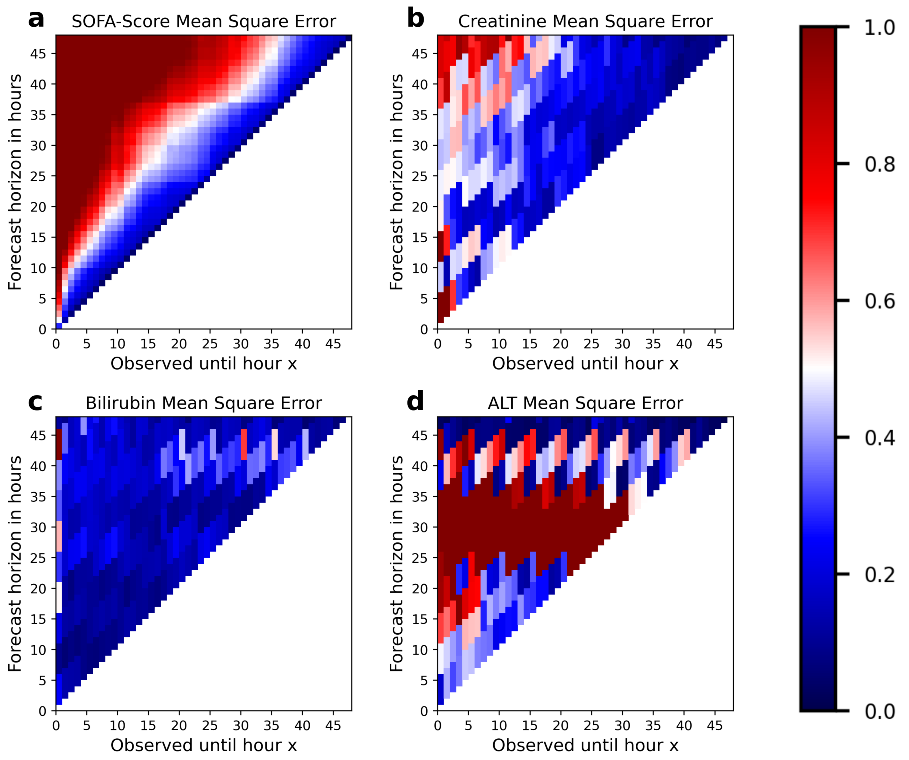

Supplementary Figure 1: **Accuracy of OptAB's forecasts of the SOFA-Score and laboratory values indicating side effects in AmsterdamUMCdb.** The heatmaps display the mean squared error (MSE) of OptAB's forecasts of the SOFA-Score (**a**), creatinine (**b**), bilirubin total (**c**) and alanine transaminase (ALT) (**d**). On both axes, time zero corresponds to Sepsis onset. The observation time on the x-axis is defined as the time span for which the patient data were assimilated into OptAB, before the forecast is made. The forecast horizon on the y-axis is the time span for which the forecast is made into the future. MSE-values are given in units of variance over all observations for the respective variable. For creatinine, bilirubin total and ALT, we averaged the MSE over 5 consecutive observation time points to account for sparser measurements.

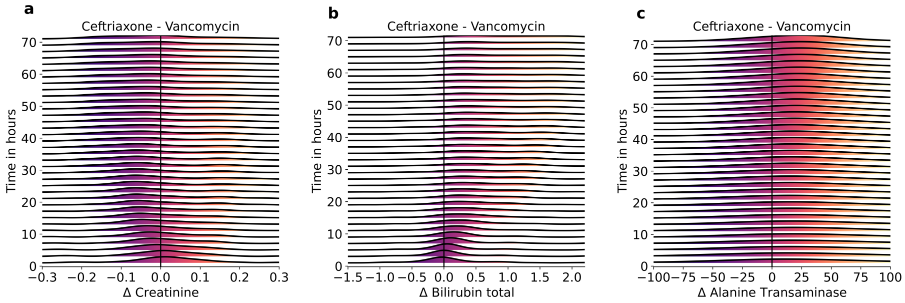

Supplementary Figure 2: **OptAB predicts plausible side effects under simulated treatments in AmsterdamUMCdb.** OptAB was used to predict laboratory values indicating side effects in response to treatment with Vancomycin versus Ceftriaxone. For each patient, the time courses of the laboratory values under both alternative treatments were predicted by OptAB for a forecast time horizon of 72 hours starting at the factual treatment initialization (time=0). Then, the difference Δ between the laboratory values under Vancomycin and Ceftriaxone treatment was taken for each patient. The density estimates indicate the temporal evolution of these differences. Density estimates for the difference of (**a**) creatinine (Δ Creatinine) values, (**b**) bilirubin total (Δ Bilirubin total) values and (**c**) alanine transaminase (Δ Alanine Transaminase) values predicted for counterfactual treatment with Vancomycin versus Ceftriaxone. Because no patient was treated with Piperacillin/Tazobactam and only 35 patients with Vancomycin, we downsampled the patients treated with Ceftriaxone to 35.

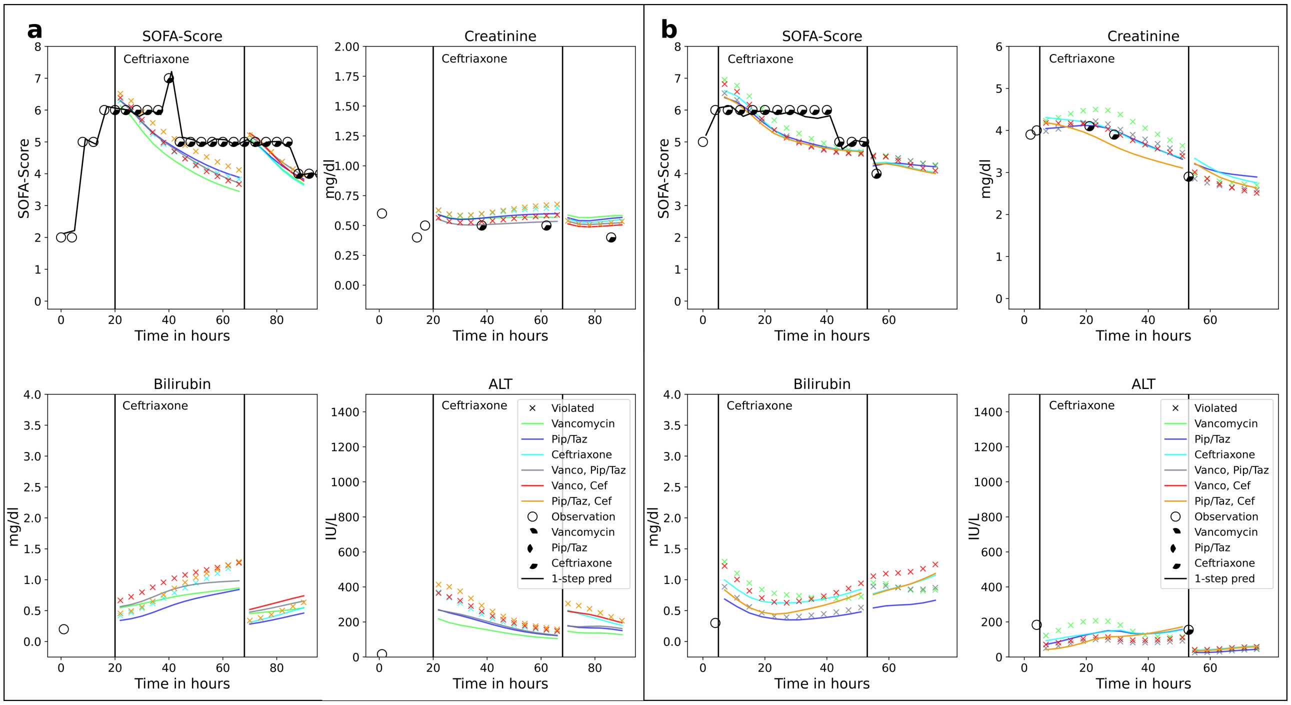

Supplementary Figure 3: **Individualized treatment effect predictions for two patients of AmsterdamUMCdb.** OptAB's predictions of the SOFA-score and side effect associated laboratory values creatinine, bilirubin total and alanine transaminase for two different patients (**a**) and (**b**). The factual data for the patients are shown as black circles which are filled to indicate the antibiotics actually received by the patient (see legend). For the SOFA score we also show the one-hour forecasts as black lines for the factual treatment. The coloured lines are the counterfactual long-term predictions for all pairwise combinations of the antibiotics Vancomycin, Piperacillin/Tazobactam and Ceftriaxone. The vertical black lines indicate the evaluation times of the treatment efficacy and the start of the next optimal antibiotic selection iteration based on further predictions of OptAB by assimilating all observed measurements up to that time. Crosses indicate that antibiotic-specific thresholds are violated for this treatment. The optimal treatment can be selected as the one with the long-term prediction (line) with the smallest SOFA-score in the treatment window at the next evaluation time point. Please note the different scales of the plots.

Detailed definition of the SOFA-Score

The Sepsis-related organ failure score (SOFA-Score) is the most important measure for assessing the severity of Sepsis. It comprises six subscores, each ranging from zero (normal) to four (abnormal) corresponding to the condition of the central nervous system, the cardiovascular system, the respiratory system, coagulation, liver and kidney (see Supplementary Table 1) [2].

**Organ**<br>*Parameter*<br>Unit	**1**	**2**	**3**	**4**
**Respiration**<br>PaO_2/FiO_2<br>mmHg	< 400	< 300	< 200	< 100
**Coagulation**<br>*Platelets*<br>/µl	< 150.000	< 100.000	< 50.000	< 20.000
**Liver**<br>*Bilirubin*<br>mg/dl	$1.2 - 1.9$	$2.0 - 5.9$	$6.0 - 11.9$	> 12.0
**Cardiovascular**<br>*MAP*<br>or *Vasopressors*<br>mmHG<br>or µg/kg/min	MAP < 70	Dopam. ≤ 5<br>or dobutam.	Dopam. > 5<br>or epineph. ≤ 0.1<br>or norepin. ≤ 0.1	Dopam. > 15<br>or epineph. > 0.1<br>or norepin. > 0.1
**Central Nervous System**<br>*GCS*	$13 - 14$	$10 - 12$	$6 - 9$	< 6
**Renal**<br>*Creatinine*<br>or *urine*<br>mg/dl<br>or ml/d	$1.2 - 1.9$	$2.0 - 3.4$	$3.5 - 4.9$<br>or urine < 500	> 5.0

Supplementary Table 1: **Definition of the SOFA-Score**. The SOFA-Score comprises six subscores, each ranging from zero to four corresponding to the condition of the central nervous system, the cardiovascular system, the respiratory system, coagulation, liver and kidney. If an organ system is not impaired according to the criteria, the subscore is set to zero.

Reporting Guidelines

The report is based on the Standards for Reporting Qualitative Research (SRQR) reporting guidelines [3].

Standards for Reporting Qualitative Research (SRQR)*	Item	Line no(s).
**Title and abstract**		
Title	Concise description of the nature and topic of the study. Identifying the study as qualitative or indicating the approach (e.g., ethnography, grounded theory) or data collection methods (e.g., interview, focus group) is recommended.	Page 1
Abstract	Summary of key elements of the study using the abstract format of the intended publication; typically includes background, purpose, methods, results, and conclusions.	Page 1-2
**Introduction**		
Problem formulation	Description and significance of the problem/phenomenon studied; review of relevant theory and empirical work; problem statement.	Lines 1-32
Purpose or research question	Purpose of the study and specific objectives or questions.	Lines 33-39
**Methods**		
Qualitative approach and research paradigm	Qualitative approach (e.g., ethnography, grounded theory, case study, phenomenology, narrative research) and guiding theory if appropriate; identifying the research paradigm (e.g., postpositivist, constructivist/interpretivist) is also recommended; rationale.	Lines 501-544

Researcher characteristics and reflexivity	Researchers' characteristics that may influence the research, including personal attributes, qualifications/experience, relationship with participants, assumptions, and/or presuppositions; potential or actual interaction between researchers' characteristics and the research questions, approach, methods, results, and/or transferability.	Lines 778-781
Context	Setting/site and salient contextual factors; rationale.	Lines 501-544
Sampling strategy	How and why research participants, documents, or events were selected; criteria for deciding when no further sampling was necessary (e.g., sampling saturation); rationale.	Lines 579-605
Ethical issues pertaining to human subjects	Documentation of approval by an appropriate ethics review board and participant consent, or explanation for lack thereof; other confidentiality and data security issues.	Lines 552-567
Data collection methods	Types of data collected; details of data collection procedures including (as appropriate) start and stop dates of data collection and analysis, iterative process, triangulation of sources/methods, and modification of procedures in response to evolving study findings; rationale.	Lines 545-551

Data collection instruments and technologies	Description of instruments (e.g., interview guides, questionnaires) and devices (e.g., audio recorders) used for data collection; if/how the instrument(s) changed over the course of the study.	No data was collected
Units of study	Number and relevant characteristics of participants, documents, or events included in the study; level of participation (could be reported in results).	Table 1
Data processing	Methods for processing data prior to and during analysis, including transcription, data entry, data management and security, verification of data integrity, data coding, and anonymization/de-identification of excerpts.	Lines 579-606
Data analysis	Process by which inferences, themes, etc., were identified and developed, including the researchers involved in data analysis; usually references a specific paradigm or approach; rationale.	Lines 501-544
Techniques to enhance trustworthiness	Techniques to enhance trustworthiness and credibility of data analysis (e.g., member checking, audit trail, triangulation); rationale.	Lines 227-266
**Results/findings**		
Synthesis and interpretation	Main findings (e.g., interpretations, inferences, and themes); might include development of a theory or model, or integration with prior research or theory.	Lines 111-398
Links to empirical data	Evidence (e.g., quotes, field notes, text excerpts, photographs) to substantiate analytic findings.	Lines 545-551

**Discussion**		
Integration with prior work, implications, transferability, and contribution(s) to the field	Short summary of main findings; explanation of how findings and conclusions connect to, support, elaborate on, or challenge conclusions of earlier scholarship; discussion of scope of application/generalizability; identification of unique contribution(s) to scholarship in a discipline or field.	Lines 399-497
Limitations	Trustworthiness and limitations of findings.	Lines 414-497
**Other**		
Conflicts of interest	Potential sources of influence or perceived influence on study conduct and conclusions; how these were managed.	Lines 782-783
Funding	Sources of funding and other support; role of funders in data collection, interpretation, and reporting.	Lines 773-777

Supplementary Table 2: Standards for Reporting Qualitative Research (SRQR) Checklist [3, p.1247,table 1]

Extended Results

In this section we present further results of OptAB using the test set from the Mimic-IV dataset [4, 5].

Limited influence of Hidden Confounders on OptAB's predictions

To illustrate our counterfactual matching approach, we present OptAB's predictions of the SOFA-score for the matched patient pair with the highest similarity (See Supplementary Figure 4). For patient **a**, treatment with the combination of Vancomycin and Piperacillin/Tazobactam was initialized one hour after Sepsis onset. The SOFA-Score of patient **a** increased from two to three at treatment initialization. Ten hours after Sepsis onset, the SOFA-Score abruptly rose from

four to ten. The creatinine level of 2.5 mg/dl at treatment initialization exceeded the acute kidney injury associated threshold for Vancomycin. OptAB captures the increasing trend of the SOFA-Score under the factual treatment of Vancomycin and Piperacillin/Tazobactam, but does not predict the abrupt detoriation to a SOFA-Score of eight at ten hours after sepsis onset. OptAB predicts a lower increase of the SOFA-Score to approximately 4.5 for treatment with Ceftriaxone. Consequently, OptAB recommends treating patient **a** with Ceftriaxone.

Patient **b** is the most similar patient to patient **a** based on covariates measured at Sepsis onset. Patient **b** also shows an increasing SOFA-Score from zero at Sepsis onset to three at treatment initialization, but was treated with Ceftriaxone instead of Vancomycin and Piperacillin/Tazobactam and did not exhibit a further abrupt detoriation in the SOFA-Score. Similar to patient **a**, the creatinine values of patient **b** exceed the acute kidney injury associated threshold for Vancomycin. Since OptAB predicts the lowest SOFA-Score for treatment with Ceftriaxone, OptAB proposes treating patient **b** with Ceftriaxone corresponding to the factual treatment received.

Patient **a** and patient **b** exhibit similar SOFA-Score dynamics until the SOFA-Score of patient **a** abruptly increases to eight at ten hours after treatment initialization. In contrast, patient **b**, who was factually treated with OptAB's proposed optimal treatment Ceftriaxone, does not show this SOFA-Score detoriation. Treatment with Vancomycin should be avoided in both patients due to elevated creatinine values indicating impaired renal function. The sharp SOFA-Score increase in patient **a** was likely induced or worsened by the administration of the contraindicated Vancomycin. This suggests that OptAB's prediction of a lower SOFA-Score increase for patient **a** is realistic and not or only slightly biased by hidden confounders.

Additional heatmaps illustrating OptAB's forecasting accuracy

We present additional heatmaps illustrating OptAB's forecasting accuracy. To ensure comparability we display the mean squared error in units of variance and cap the scale at 1 in the heatmaps of the main text. Here, we present heatmaps that include the maximum observed MSE in units of variance for each variable. (see Supplementary Figures 5 and 6).

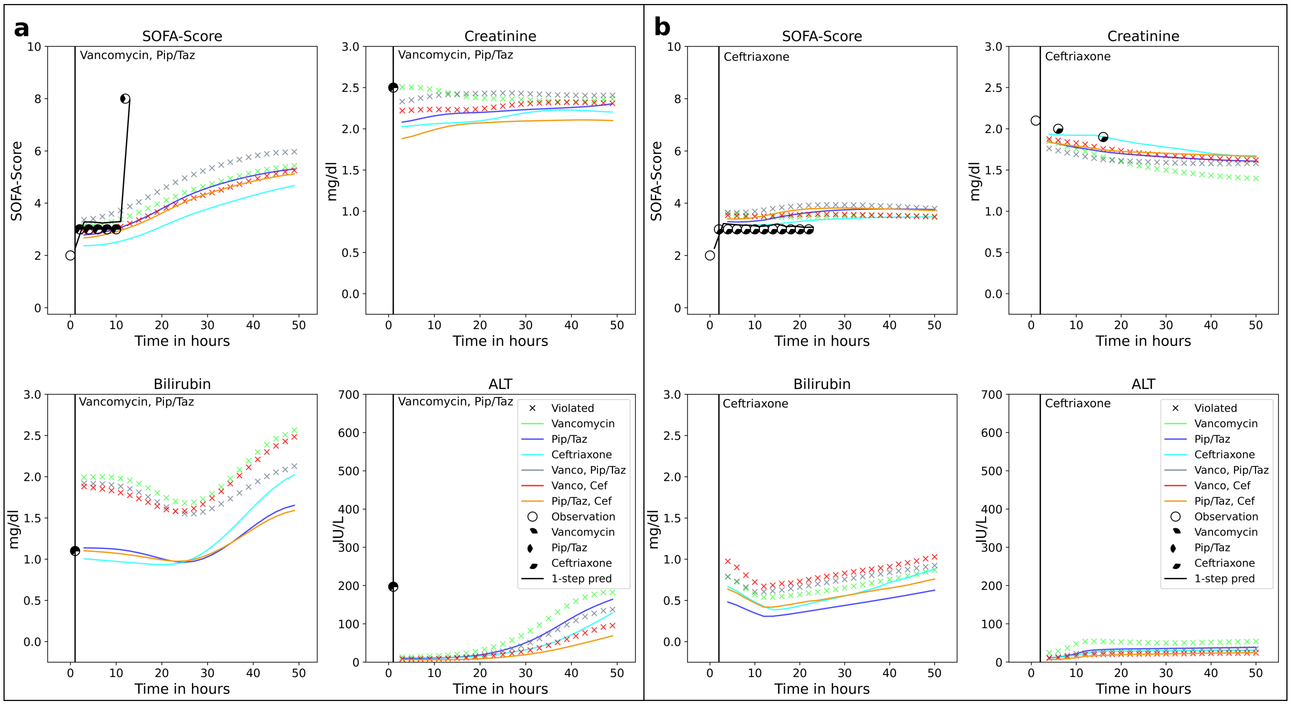

Supplementary Figure 4: **OptAB's disease progression predictions for a matched patient pair.** The figure shows OptAB's predictions of the SOFA-score and the laboratory values creatinine, bilirubin total and alanine transaminase for the counterfactual matched patient pair with the highest similarity. Plot (**b**) shows the patient who is most similar to the patient shown in plot (**a**). The factual data for the patients are shown as black circles which are filled to indicate the antibiotics actually received by the patient (see legend). For the SOFA score we also show the one-hour forecasts as black lines for the factual treatment. The coloured lines are the counterfactual long-term predictions for all pairwise combinations of the antibiotics Vancomycin, Piperacillin/Tazobactam and Ceftriaxone. The vertical black lines indicate the start of the first optimal antibiotic selection iteration based on all observed measurements up to that time. Crosses indicate that antibiotic-specific thresholds are violated for this treatment. The optimal treatment can be selected as the one with the long-term prediction (line) with the smallest SOFA-score in the treatment window at the next evaluation time point.

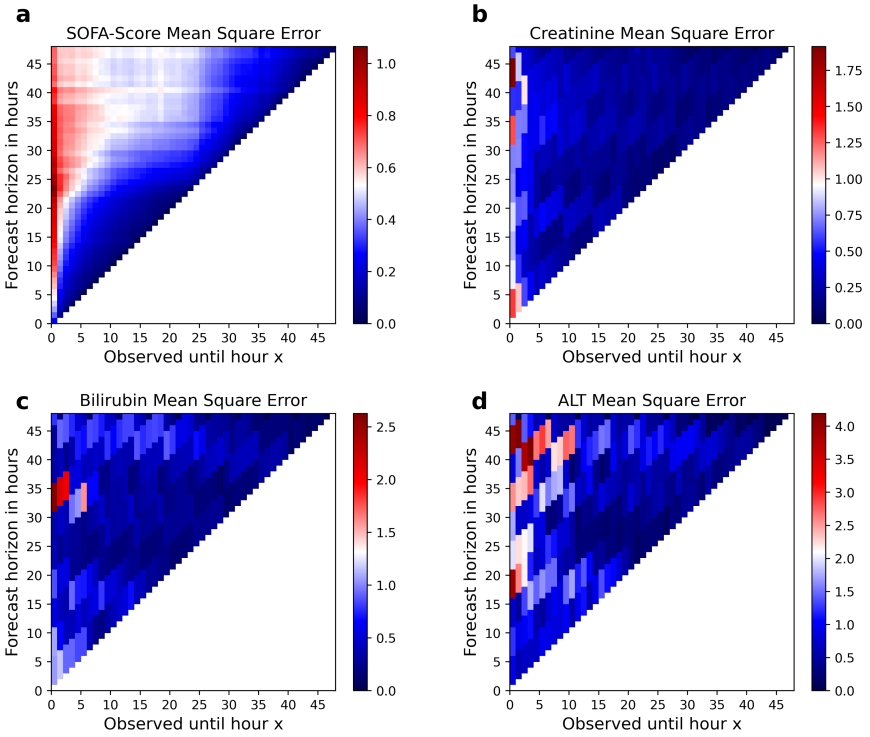

Supplementary Figure 5: **Additional heatmaps illustrating OptAB's forecasting abilities for the MIMIC-IV dataset including the maximum observed MSE for each variable**. The heatmaps display the mean squared error (MSE) of OptAB's forecasts of the SOFA-Score (**a**), creatinine (**b**), bilirubin total (**c**) and alanine transaminase (ALT) (**d**). On both axes, time zero corresponds to Sepsis onset. The observation time on the x-axis is defined as the time span for which the patient data were assimilated into OptAB, before the forecast is made. The forecast horizon on the y-axis is the time span for which the forecast is made into the future. MSE-values are given in units of variance over all observations for the respective variable. For creatinine, bilirubin total and ALT, we averaged the MSE over 5 consecutive observation time points to account for sparser measurements. In the main text, heatmaps were presented with a capped scale at 1 to ensure comparability. Here, the heatmaps include the maximum observed MSE in units of variance for each variable.

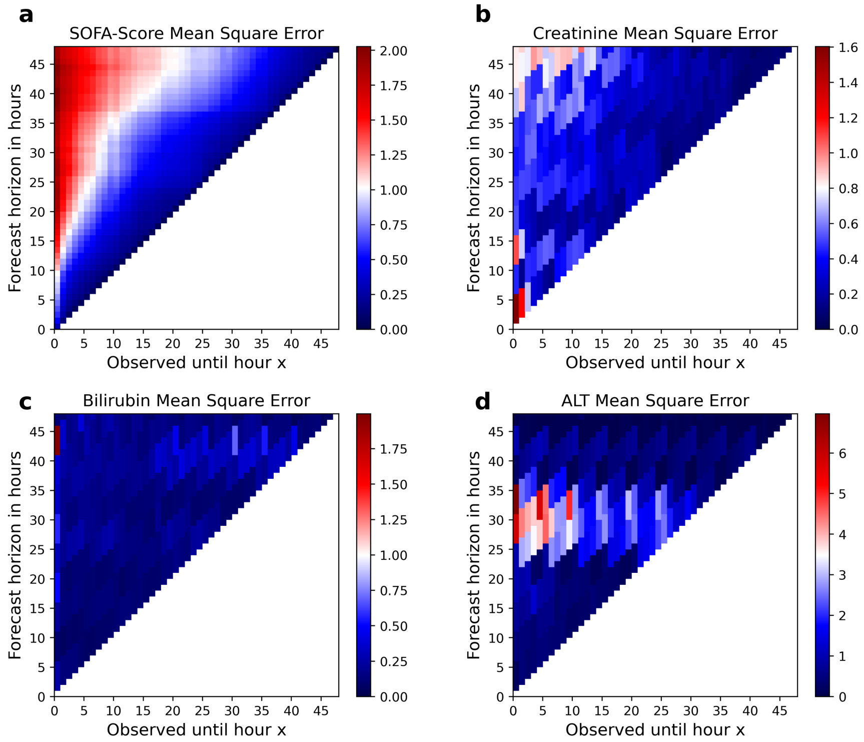

Supplementary Figure 6: **Additional heatmaps illustrating OptAB's forecasting abilities for AmsterdamUMCdb including the maximum observed MSE for each variable**. The heatmaps display the mean squared error (MSE) of OptAB's forecasts of the SOFA-Score (**a**), creatinine (**b**), bilirubin total (**c**) and alanine transaminase (ALT) (**d**). On both axes, time zero corresponds to Sepsis onset. The observation time on the x-axis is defined as the time span for which the patient data were assimilated into OptAB, before the forecast is made. The forecast horizon on the y-axis is the time span for which the forecast is made into the future. MSE-values are given in units of variance over all observations for the respective variable. For creatinine, bilirubin total and ALT, we averaged the MSE over 5 consecutive observation time points to account for sparser measurements. In the main text, heatmaps were presented with a capped scale at 1 to ensure comparability. Here, the heatmaps include the maximum observed MSE in units of variance for each variable.

Additional example patients for OptAB's optimal antibiotic selection

In this section we present two additional example patients from the MIMIC-IV testset to illustrate OptAB's optimal treatment selection. (see Supp. Fig. 7).

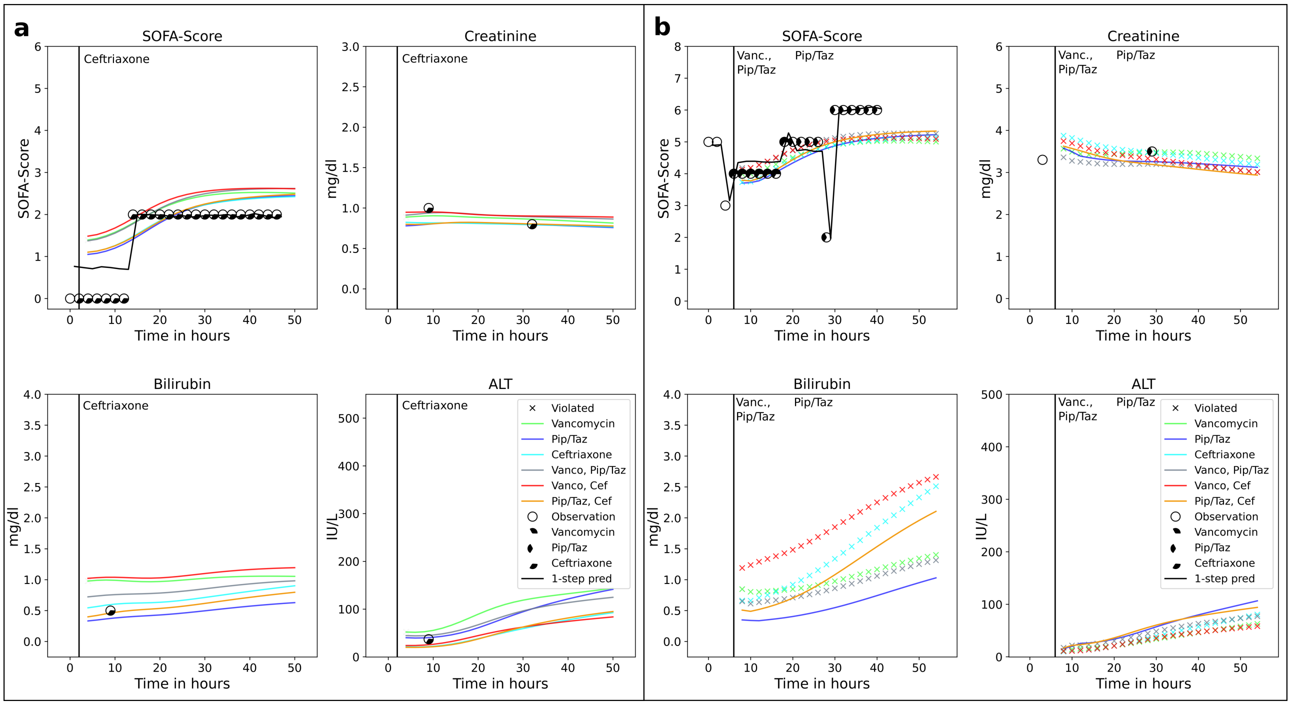

Supplementary Figure 7: **Individualized treatment effect predictions for two additional example patients.** OptAB's predictions of the SOFA-score and side effect associated laboratory values creatinine, bilirubin total and alanine transaminase for two different patients (**a**) and (**b**). The factual data for the patients are shown as black circles which are filled to indicate the antibiotics actually received by the patient (see legend). For the SOFA score we also show the one-hour forecasts as black lines for the factual treatment. The coloured lines are the counterfactual long-term predictions for all pairwise combinations of the antibiotics Vancomycin, Piperacillin/Tazobactam and Ceftriaxone. The vertical black lines indicate the evaluation times of the treatment efficacy and the start of the next optimal antibiotic selection iteration based on further predictions of OptAB by assimilating all observed measurements up to that time. Crosses indicate that antibiotic-specific thresholds are violated for this treatment. The optimal treatment can be selected as the one with the long-term prediction (line) with the smallest SOFA-score in the treatment window at the next evaluation time point. Please note the different scales of the plots.

For Supplementary example patient **a**, treatment with Ceftriaxone is initialized 2 hours after Sepsis onset. The patient's disease course seems relatively mild, starting with a SOFA-Score of 0 at Sepsis onset, which increases to 2 at

13 hours after Sepsis onset.

OptAB's one-hour forecasts and its 48-hour forecast under factual treatment accurately capture the increasing dynamic of the SOFA-Score, although OptAB overestimates the SOFA-Score by 1 at treatment initialization. The counterfactual SOFA-Score forecasts for treatment with Piperacillin/Tazobactam and Ceftriaxone exhibit a similar dynamic. OptAB's forecasts a slightly higher SOFA-Score for treatments including Vancomycin.

OptAB accurately forecasts the observed creatinine value of 0.8 mg/dl 32 hours after Sepsis onset under Ceftriaxone treatment, but slightly underestimates the creatinine value of 1 mg/dl at 8 hours after Sepsis onset. The counterfactual creatinine forecasts are 0.15 mg/dl higher for treatments including the nephrotoxic Vancomycin. OptAB accurately forecasts the measured values of bilirubin total and alanine transaminase at 9 hours after Sepsis for the factual treatment with Ceftriaxone. Although the forecasts of these variables under counterfactual treatments vary, all forecasts remain below the side-effects associated thresholds.

For supplementary example patient **a**, OptAB accurately forecasts the SOFA-Score and the laboratory values indicating side-effects. OptAB recommends treatment with Ceftriaxone or Piperacillin/Tazobactam, as both treatments lead to similarly low SOFA-Scores without side-effects or contraindications.

For supplementary example patient **b**, treatment with the combination of Piperacillin/Tazobactam and Vancomycin was initialized 6 hours after Sepsis onset and was switched to Piperacillin/Tazobactam at 19 hours after Sepsis onset.

With the exception of a brief drop 28 hours after sepsis onset, the SOFA score continuously increases from 4 at treatment start to 6. OptAB correctly forecasts the increasing trend of the SOFA-Score for the factual treatment of Vancomycin and Piperacillin/Tazobactam, but slightly underestimates the increase to 6 at 30 hours after Sepsis onset. OptAB's counterfactual forecasts of the SOFA-Score show a similar dynamic.

The creatinine levels of Supplementary example patient **b** are already elevated at 2 hours after Sepsis onset increase and increase further to 3.5 mg/dl 28 hours after Sepsis onset. OptAB slightly underestimates the creatinine value of 3.5 mg/dl at 28 hours after Sepsis onset for the factual treatment. OptAB's counterfactual forecasts exhibit similar dynamics.

Unfortunately, no measurements of bilirubin total and alanine transaminase are available for Supplementary example patient **b**. However, the forecasts of

alanine transaminase show similar dynamics for all treatments. Conversely, the forecasts for bilirubin total differ. The bilirubin total forecasts under treatments including the hepatotoxic Ceftriaxone demonstrate a much steeper increase compared to OptAB's forecasts for treatments excluding Ceftriaxone.

Presumably, the factual treatment with the nephrotoxic Vancomycin was discontinued due to elevated and increasing creatinine levels. For Supplementary example patient **b** OptAB recommends treatment with Piperacillin/Tazobactam instead of the combination of Vancomycin and Piperacillin/Tazobactam due to elevated creatinine levels and warns against treatment with the contraindicated Vancomycin and Ceftriaxone.

Additional plots illustrating OptAB's treatment improvement

In this section we present additional plots illustrating OptAB's average treatment improvement. We present plots of the mean reduction of the SOFA-Score for additional treatment iterations up to the last timepoint where more than 5 % of the patients remained in the Intensive Care Unit. We considered two different scenarios: (a) Selection of the optimal treatment under the constraints imposed by side effects and contraindications and (b) minimal SOFA-score regardless of side effects (see Supplementary Figure 8). Caution is required when interpreting these results, as OptAB's treatment optimization for iterations beyond the first one relies on observation data under the factual treatment, which may introduce biases in treatment effect estimation of the optimal treatment. The mean reduction in the SOFA-Score for OptAB's optimal treatment during the first treatment iteration slightly differ from the plots presented in the main text due to treatment changes during the first 48 hours triggered by contraindications or blood cultures.

Hyperparameter optimization for OptAB

In this section we present the search spaces of the hyperparameters of OptAB including the final hyperparameterconfigurations for the Encoder and the Decoder (see Supplementary Table 3). The hyperparameteroptimization of the Decoder was initialized with the final hyperparameters of the Encoder.

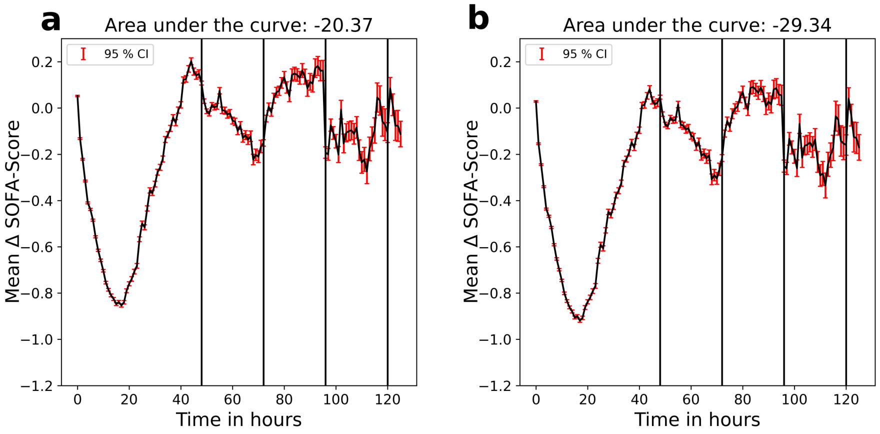

Supplementary Figure 8: **Estimated treatment effect for the optimal antibiotics compared to the factual treatment.** This figure presents the average difference of the SOFA-score for the optimal treatment according to OptAB's predictions relative to the factual treatment up to the last timepoint when more than 5 % of the patients remained in the Intensive Care Unit. The optimal treatment was defined as the antibiotic(s) combination leading to the lowest SOFA-score after the consecutive treatment phases of 48 and 24 hours. The bars indicate 95%-confidence intervals. In (**a**) the side-effects and contraindications were taken into account, whereas in (**b**) the treatment with the highest reduction of the SOFA-Score regardless of the side effects was selected. The area under the curve indicates the treatment effect integrated over time. The vertical black line indicates the start of the next optimal antibiotic selection iteration of OptAB.

**Hyperparameter**	**Final values (Encoder)**	**Final values (Decoder)**	**Search Space**
Batch size	500	1000	$\{100, 200, 500, 1000, 2000\}$
Learning rate	0.00507	0.00507	$[0.0001, 0.01]$
Dimension P of the latent state	17	17	$\{1, 2, ..., 30\}$
Number of layers of F	15	15	$\{1, 2, ..., 20\}$
Maximum number of units + P of F	33	33	$\{1, 2, ..., 1000\}$
Activation function of F	tanh	tanh	$\{\text{leakyrelu}, \text{tanh}, \text{sigmoid}, \text{identity}\}$
Maximum number of units of h	1	1	$\{1, 2, ..., 6\}$
Number of layers of h	128	128	$\{1, 2, ..., 1000\}$
Activation function of h	tanh	tanh	$\{\text{leakyrelu}, \text{tanh}, \text{sigmoid}, \text{identity}\}$

Supplementary Table 3: **Final hyperparameterconfigurations of OptAB**. Table presenting the search spaces of the hyperparameters of OptAB including the final hyperparameterconfigurations for the Encoder and the Decoder.

Supplementary References

[1] Thoral, P. J. *et al.* Sharing ICU Patient Data Responsibly Under the Society of Critical Care Medicine/European Society of Intensive Care Medicine Joint Data Science Collaboration: The Amsterdam University Medical Centers Database (AmsterdamUMCdb) Example. *Crit. Care Med.* **49**, e563–e577 (2021).

[2] Vincent, J. L. *et al.* The SOFA (Sepsis-related Organ Failure Assessment) score to describe organ dysfunction/failure: On behalf of the Working Group on Sepsis-Related Problems of the European Society of Intensive Care Medicine. *Intensive Care Med.* **22**, 707–710 (1996).

[3] O'Brien, B. C., Harris, I. B., Beckman, T. J., Reed, D. A. & Cook, D. A. Standards for Reporting Qualitative Research: A Synthesis of Recommendations. *Acad. Med.* **89**, 1245–1251 (2014).

[4] Johnson, A. E. W. *et al.* MIMIC-IV, a freely accessible electronic health record dataset. *Sci. Data* **10**, 1 (2023).

[5] Goldberger, A. L. *et al.* PhysioBank, PhysioToolkit, and PhysioNet: Components of a New Research Resource for Complex Physiologic Signals. *Circulation* **101**, E215–E220 (2000).
